# Supplementary material for: Mendelian randomization accounting for complex correlated horizontal pleiotropy while elucidating shared genetic etiology
Source: Nat Commun. 2022 Oct 30;13:6490. doi: 10.1038/s41467-022-34164-1 (PMC9618026; doi:10.1038/s41467-022-34164-1)
Supplement: Supplementary file 1 — Supplementary Information [file 41467_2022_34164_MOESM1_ESM.pdf]

Supplementary Materials for "Mendelian randomization accounting for complex correlated horizontal pleiotropy while elucidating shared genetic etiology"

Qing Cheng[1,2], Xiao Zhang[2], Lin S. Chen[3*], Jin Liu[2*]

[1] Center of Statistical Research, School of Statistics, Southwestern University of Finance and Economics, Chengdu, Sichuan, China

[2]Centre for Quantitative Medicine, Health Services & Systems Research, Duke-NUS Medical School

[3]Department of Public Health Sciences, The University of Chicago

[*]Corresponding authors. E-mails: lchen@health.bsd.uchicago.edu; jin.liu@duke-nus.edu.sg;

Supplementary Notes

Supplementary Note 1. The Gibbs sampler algorithms for MR-CUE estimation 3

1.1 For independent SNPs 3

1.2 For correlated SNPs 5

Supplementary Note 2. Pseudo-code 8

Supplementary Note 3. Additional simulation results 13

3.1 The identifiability of causal effect 13

3.2 Simulation results for different p and $\rho_{\alpha\gamma}$ 15

3.3 Generation of summary statistics for multiple confounding pathways 25

3.4 Simulation results for non-linear confounders 26

3.5 Simulation results for binary outcome 27

3.6 Simulation results for different proportions of IVs with CHP effects 27

3.7 Simulation results for sparse θ in reverse causation analysis 28

Supplementary Note 4. Sensitive Analysis 29

4.1 Negative and Positive Controls 29

Supplementary Note 5. Real Data Analysis 32

Supplementary References 35

Supplementary Note 1
The Gibbs sampler algorithms for MR-CUE estimation

In this section, we present the details on deriving Gibbs sampler for MR-CUE.

1.1 For independent SNPs

We propose the following hierarchical Bayesian model for independent SNPs.

$$
\begin{aligned}
&\left(\begin{array}{c}\widehat{\gamma}_k\\ \widehat{\Gamma}_k\end{array}\right)\sim\mathcal{N}\left(\left(\begin{array}{c}\gamma_k\\ \Gamma_k\end{array}\right),\left(\begin{array}{cc}\widehat{s}^2_{\boldsymbol{\gamma}_k}, & \widehat{\rho}_e\widehat{s}_{\boldsymbol{\gamma}_k}\widehat{s}_{\boldsymbol{\Gamma}_k}\\ \widehat{\rho}_e\widehat{s}_{\boldsymbol{\gamma}_k}\widehat{s}_{\boldsymbol{\Gamma}_k}, & \widehat{s}^2_{\boldsymbol{\Gamma}_k}\end{array}\right)\right),\\
&\gamma_k|\sigma^2_\gamma\overset{i.i.d.}{\sim}\mathcal{N}(0,\sigma^2_\gamma),\quad \Gamma_k|\beta_1,\beta_2,\gamma_k,\eta_k,\tau_1^2,\tau_2^2,\xi^2\overset{i.i.d.}{\sim}\left\{\mathcal{N}(\beta_1\gamma_k,\xi^2\tau_1^2)\right\}^{(1-\eta_k)}\left\{\mathcal{N}(\beta_2\gamma_k,\tau_2^2)\right\}^{\eta_k},\\
&\sigma^2_\gamma\sim\mathcal{IG}(a_\gamma,b_\gamma),\quad \tau_1^2\sim\mathcal{IG}(a_{\tau1},b_{\tau1}),\quad \tau_2^2\sim\mathcal{IG}(a_{\tau2},b_{\tau2}),\\
&\Pr(\xi^2)\propto\tfrac{1}{\xi^2},\quad \eta_k|\omega\overset{i.i.d.}{\sim}\omega^{\eta_k}(1-\omega)^{1-\eta_k},\quad \omega\sim Beta(a,b).
\end{aligned}
\tag{1}
$$

Based on the Bayesian model (1), the joint posterior density of all the latent variables is

$$
\begin{aligned}
&\Pr(\boldsymbol{\Gamma},\boldsymbol{\gamma},\sigma^2_{\boldsymbol{\gamma}},\tau_1^2,\tau_2^2,\xi^2,\boldsymbol{\eta},\omega|\widehat{\boldsymbol{\Gamma}},\widehat{\boldsymbol{\gamma}})\\
\propto\;&\Pr(\widehat{\boldsymbol{\Gamma}},\widehat{\boldsymbol{\gamma}}|\boldsymbol{\Gamma},\boldsymbol{\gamma})\Pr(\boldsymbol{\Gamma}|\beta_1,\beta_2,\boldsymbol{\gamma},\boldsymbol{\eta},\tau_1^2,\tau_2^2,\xi^2)\Pr(\boldsymbol{\gamma}|\sigma^2_{\boldsymbol{\gamma}})\Pr(\boldsymbol{\eta}|\omega)\Pr(\xi^2)\Pr(\sigma_{\boldsymbol{\gamma}})\Pr(\tau_1^2)\Pr(\tau_2^2)\Pr(\omega)\\
=\;&\prod_{k=1}^{p}\left\{\Pr(\widehat{\Gamma}_k,\widehat{\gamma}_k|\Gamma_k,\gamma_k)\Pr(\Gamma_k|\beta_1,\beta_2,\gamma_k,\eta_k,\tau_1^2,\tau_2^2,\xi^2)\Pr(\gamma_k|\sigma^2_{\boldsymbol{\gamma}})\Pr(\eta_k|\omega)\right\}\\
&\Pr(\xi^2)\Pr(\sigma_{\boldsymbol{\gamma}})\Pr(\tau_1^2)\Pr(\tau_2^2)\Pr(\omega)
\end{aligned}
$$

The factors in the joint posterior density that involve Γ_k form a mixture of two normal distributions. The conditional posterior distribution of each Γ_k given the other parameters in the model is

$$
\Gamma_k|\widehat{\Gamma}_k,\gamma_k,\eta_k,\beta_1,\beta_2,\tau_1^2,\tau_2^2,\xi^2\sim(1-\eta_k)\,\mathcal{N}(\widetilde{\mu}_{k0},\widetilde{\sigma}^2_{k0})+\eta_k\,\mathcal{N}(\widetilde{\mu}_{k1},\widetilde{\sigma}^2_{k1}),\tag{2}
$$

where $(\widetilde{\mu}_{k0}, \widetilde{\sigma}^2_{k0})$ and $(\widetilde{\mu}_{k1}, \widetilde{\sigma}^2_{k1})$ satisfy

$$\begin{cases} -\frac{1}{2\widetilde{\sigma}^2_{k0}} = -\frac{1}{2}\left(\frac{1}{(1-\widehat{\rho}^2_e)\widehat{s}^2_{\Gamma_k}} + \frac{1}{\xi^2\tau_1^2}\right), \\ \frac{\widetilde{\mu}_{k0}}{\widetilde{\sigma}^2_{k0}} = \frac{\widehat{\Gamma}_k}{(1-\widehat{\rho}^2_e)\widehat{s}^2_{\Gamma_k}} + \frac{\widehat{\rho}_e(\gamma_k - \widehat{\gamma}_k)}{(1-\widehat{\rho}^2_e)\widehat{s}_{\gamma_k}\widehat{s}_{\Gamma_k}} + \frac{\beta_1\gamma_k}{\xi^2\tau_1^2}, \end{cases} \quad \text{and} \quad \begin{cases} -\frac{1}{2\widetilde{\sigma}^2_{k1}} = -\frac{1}{2}\left(\frac{1}{(1-\widehat{\rho}^2_e)\widehat{s}^2_{\Gamma_k}} + \frac{1}{\tau_2^2}\right), \\ \frac{\widetilde{\mu}_{k1}}{\widetilde{\sigma}^2_{k1}} = \frac{\widehat{\Gamma}_k}{(1-\widehat{\rho}^2_e)\widehat{s}^2_{\Gamma_k}} + \frac{\widehat{\rho}_e(\gamma_k - \widehat{\gamma}_k)}{(1-\widehat{\rho}^2_e)\widehat{s}_{\gamma_k}\widehat{s}_{\Gamma_k}} + \frac{\beta_2\gamma_k}{\tau_2^2}. \end{cases}$$

Conditioning on the data $(\widehat{\boldsymbol{\Gamma}}, \widehat{\boldsymbol{\gamma}})$ and other parameters, the conditional distribution for each element γ_k comes from a mixture of two normal distributions, i.e.,

$$\gamma_k|\widehat{\gamma}_k, \Gamma_k, \eta_k, \beta_1, \beta_2, \tau_1^2, \tau_2^2, \sigma^2_\gamma, \xi^2 \sim (1-\eta_k)\ \mathcal{N}(\mu_{k0}, \sigma^2_{k0}) + \eta_k\ \mathcal{N}(\mu_{k1}, \sigma^2_{k1}), \tag{3}$$

where $(\mu_{k0}, \sigma^2_{k0})$ and $(\mu_{k1}, \sigma^2_{k1})$ satisfy

$$\begin{cases} -\frac{1}{2\sigma^2_{k0}} = -\frac{1}{2}\left(\frac{1}{(1-\widehat{\rho}^2_e)\widehat{s}^2_{\gamma_k}} + \beta_1^2\frac{1}{\xi^2\tau_1^2} + \frac{1}{\sigma^2_\gamma}\right), \\ \frac{\mu_{k0}}{\sigma^2_{k0}} = \frac{\widehat{\gamma}_k}{(1-\widehat{\rho}^2_e)\widehat{s}^2_{\gamma_k}} + \frac{\widehat{\rho}_e(\Gamma_k - \widehat{\Gamma}_k)}{(1-\widehat{\rho}^2_e)\widehat{s}_{\gamma_k}\widehat{s}_{\Gamma_k}} + \frac{\beta_1\Gamma_k}{\xi^2\tau_1^2}, \end{cases} \quad \text{and} \quad \begin{cases} -\frac{1}{2\sigma^2_{k1}} = -\frac{1}{2}\left(\frac{1}{(1-\widehat{\rho}^2_e)\widehat{s}^2_{\gamma_k}} + \beta_2^2\frac{1}{\tau_2^2} + \frac{1}{\sigma^2_\gamma}\right), \\ \frac{\mu_{k1}}{\sigma^2_{k1}} = \frac{\widehat{\gamma}_k}{(1-\widehat{\rho}^2_e)\widehat{s}^2_{\gamma_k}} + \frac{\widehat{\rho}_e(\Gamma_k - \widehat{\Gamma}_k)}{(1-\widehat{\rho}^2_e)\widehat{s}_{\gamma_k}\widehat{s}_{\Gamma_k}} + \frac{\beta_2\Gamma_k}{\tau_2^2}. \end{cases}$$

The conditional posterior distributions of β_1 and β_2 are both from normal, i.e.,

$$\beta_1|\boldsymbol{\Gamma}, \boldsymbol{\gamma}, \boldsymbol{\eta}, \tau_1^2, \xi^2 \sim\ \mathcal{N}(\mu_{\beta 0}, \sigma^2_{\beta 0}), \quad \text{and} \quad \beta_2|\boldsymbol{\Gamma}, \boldsymbol{\gamma}, \boldsymbol{\eta}, \tau_2^2 \sim\ \mathcal{N}(\mu_{\beta 1}, \sigma^2_{\beta 1}), \tag{4}$$

where

$$\begin{cases} -\frac{1}{2\sigma^2_{\beta 0}} = -\frac{\sum_{k=1}^p (1-\eta_k)\gamma_k^2}{2\xi^2\tau_1^2}, \\ \frac{\mu_{\beta 0}}{\sigma^2_{\beta 0}} = \frac{\sum_{k=1}^p (1-\eta_k)\Gamma_k\gamma_k}{\xi^2\tau_1^2}, \end{cases} \quad \text{and} \quad \begin{cases} -\frac{1}{2\sigma^2_{\beta 1}} = -\frac{\sum_{k=1}^p \eta_k\gamma_k^2}{2\tau_2^2}, \\ \frac{\mu_{\beta 1}}{\sigma^2_{\beta 1}} = \frac{\sum_{k=1}^p \eta_k\Gamma_k\gamma_k}{\tau_2^2}, \end{cases}$$

Conditioning on the data and the other parameters in the model, the conditional posterior distributions of $\sigma^2_{\boldsymbol{\gamma}}$, τ_1^2,τ_2^2 , ξ^2 are all inverse-gamma, i.e.,

$$\begin{aligned} &\sigma^2_{\boldsymbol{\gamma}}|\boldsymbol{\gamma}, a_{\boldsymbol{\gamma}}, b_{\boldsymbol{\gamma}} \sim \mathcal{IG}(\widetilde{a}_{\boldsymbol{\gamma}}, \widetilde{b}_{\boldsymbol{\gamma}}), \qquad \tau_1^2|\boldsymbol{\Gamma}, \boldsymbol{\gamma}, \xi^2, a_{\tau 1}, b_{\tau 1} \sim \mathcal{IG}(\widetilde{a}_{\tau 1}, \widetilde{b}_{\tau 1}), \\ &\tau_2^2|\boldsymbol{\Gamma}, \boldsymbol{\gamma}, a_{\tau 2}, b_{\tau 2} \sim \mathcal{IG}(\widetilde{a}_{\tau 2}, \widetilde{b}_{\tau 2}), \quad \xi^2|\boldsymbol{\Gamma}, \boldsymbol{\gamma}, \beta_1, \boldsymbol{\eta} \sim \mathcal{IG}(a_\xi, b_\xi). \end{aligned} \tag{5}$$

where $\widetilde{a}_{\boldsymbol{\gamma}} = a_{\boldsymbol{\gamma}} + \frac{p}{2}$, $\widetilde{b}_{\boldsymbol{\gamma}} = b_{\boldsymbol{\gamma}} + \frac{\boldsymbol{\gamma}^{\mathrm{T}}\boldsymbol{\gamma}}{2}$, $\widetilde{a}_{\tau 1} = a_{\tau 1} + \frac{\sum_{k=1}^p (1-\eta_k)}{2}$, $\widetilde{b}_{\tau 1} = b_{\tau 1} + \frac{\sum_{k=1}^p (1-\eta_k)(\Gamma_k - \beta_1\gamma_k)^2}{2\xi^2}$, $\widetilde{a}_{\tau 2} = a_{\tau 2} + \frac{\sum_{k=1}^p \eta_k}{2}$, $\widetilde{b}_{\tau 2} = b_{\tau 2} + \frac{\sum_{k=1}^p \eta_k(\Gamma_k - \beta_2\gamma_k)^2}{2}$, $a_\xi = \frac{1}{2}\sum_{k=1}^p (1-\eta_k)$ and $b_\xi = \frac{1}{2\tau_1^2}\sum_{k=1}^p (1-\eta_k)(\Gamma_k - \beta_1\gamma_k)^2$.

The conditional posterior of ω is also a Beta distribution:

$$\omega|\boldsymbol{\eta}, a, b \sim \text{Beta}\left(a + \sum_{k=1}^{p} \eta_k, b + \sum_{k=1}^{p}(1-\eta_k)\right). \tag{6}$$

The conditional probability of $\boldsymbol{\eta}$ given $\boldsymbol{\Gamma}$ can be found using Bayes' theorem

$$\Pr(\eta_k = 1|\Gamma_k) = \frac{\Pr(\eta_k = 1)\Pr(\Gamma_k|\eta_k = 1)}{\Pr(\eta_k = 0)\Pr(\Gamma_k|\eta_k = 0) + \Pr(\eta_k = 1)\Pr(\Gamma_k|\eta_k = 1)} \tag{7}$$

Algorithm 1 The Gibbs sampler algorithm for independent SNPs

```
Initialize:

repeat
    for k = 1 to p do
        Sample Γ_k using (2).
        Sample γ_k using (3).
        Sample η_k using (7).
    end for
    Sample β_1 and β_2 using (4).
    Sample σ_γ^2, τ_1^2 , τ_2^2, ξ^2 using (5).
    Sample ω using (6).
until Maximum iteration reached
```

1.2 For correlated SNPs

For correlated SNPs, we consider the following hierarchical Bayesian model for the l-block, $l = 1, \cdots, L.$:

$$\begin{pmatrix} \widehat{\boldsymbol{\gamma}}_l \\ \widehat{\boldsymbol{\Gamma}}_l \end{pmatrix} \sim \mathcal{N}\left(\begin{pmatrix} \widehat{\mathbf{S}}_{\boldsymbol{\gamma}_l}\widehat{\mathbf{R}}^{(l)}\widehat{\mathbf{S}}_{\boldsymbol{\gamma}_l}^{-1}\boldsymbol{\gamma}_l \\ \widehat{\mathbf{S}}_{\boldsymbol{\Gamma}_l}\widehat{\mathbf{R}}^{(l)}\widehat{\mathbf{S}}_{\boldsymbol{\Gamma}_l}^{-1}\boldsymbol{\Gamma}_l \end{pmatrix}, \begin{pmatrix} \widehat{\mathbf{S}}_{\boldsymbol{\gamma}_l}, & \mathbf{0} \\ \mathbf{0}, & \widehat{\mathbf{S}}_{\boldsymbol{\Gamma}_l} \end{pmatrix} (\mathbf{R}_e \otimes \widehat{\mathbf{R}}^{(l)}) \begin{pmatrix} \widehat{\mathbf{S}}_{\boldsymbol{\gamma}_l}, & \mathbf{0} \\ \mathbf{0}, & \widehat{\mathbf{S}}_{\boldsymbol{\Gamma}_l} \end{pmatrix}\right), \tag{8}$$

$$\Gamma_{lk}|\beta_1, \beta_2, \gamma_{lk}, \eta_l, \tau_1^2, \tau_2^2, \xi^2 \overset{i.i.d.}{\sim} \left\{\mathcal{N}(\beta_1\gamma_{lk}, \xi^2\tau_1^2)\right\}^{(1-\eta_l)} \left\{\mathcal{N}(\beta_2\gamma_{lk}, \tau_2^2)\right\}^{\eta_l},$$

$$\gamma_{lk}|\sigma_\gamma^2 \overset{iid}{\sim} \mathcal{N}(0, \sigma_\gamma^2), \quad \eta_l|\omega \overset{iid}{\sim} \omega^{\eta_l}(1-\omega)^{1-\eta_l},$$

$$\sigma_\gamma^2 \sim \mathcal{IG}(a_\gamma, b_\gamma), \quad \tau_1^2 \sim \mathcal{IG}(a_{\tau 1}, b_{\tau 1}), \quad \tau_2^2 \sim \mathcal{IG}(a_{\tau 2}, b_{\tau 2}),$$

$$\Pr(\xi^2) \propto \tfrac{1}{\xi^2}, \quad \omega \sim Beta(a, b).$$

Based on model (8), the joint posterior density of all the parameters can be written as follows,

$$
\begin{aligned}
& \Pr(\boldsymbol{\Gamma}, \boldsymbol{\eta}, \beta_1, \beta_2, \tau_1^2, \tau_2^2, \xi^2 | \widehat{\boldsymbol{\Gamma}}, \widehat{\boldsymbol{\gamma}}) \\
\propto\ & \Pr(\widehat{\boldsymbol{\Gamma}}, \widehat{\boldsymbol{\gamma}} | \boldsymbol{\Gamma}, \boldsymbol{\gamma}) \Pr(\boldsymbol{\Gamma} | \beta_1, \beta_2, \boldsymbol{\gamma}, \boldsymbol{\eta}, \tau_1^2, \tau_2^2, \xi^2) \Pr(\boldsymbol{\gamma} | \sigma_{\boldsymbol{\gamma}}^2) \Pr(\boldsymbol{\eta} | \omega) \Pr(\xi^2) \Pr(\sigma_{\boldsymbol{\gamma}}) \Pr(\tau_1^2) \Pr(\tau_2^2) \Pr(\omega) \\
=\ & \prod_{l=1}^{L} \left[\Pr(\widehat{\boldsymbol{\Gamma}}_l, \widehat{\boldsymbol{\gamma}}_l | \boldsymbol{\Gamma}_l, \boldsymbol{\gamma}_l) \Pr(\eta_l | \omega) \Pr \left\{ \sum_{k=1}^{p_l} \Pr(\Gamma_{lk} | \beta_1, \beta_2, \gamma_{lk}, \eta_l, \tau_1^2, \tau_2^2, \xi^2) \Pr(\gamma_{lk} | \sigma_{\boldsymbol{\gamma}}^2) \right\} \right] \\
& \Pr(\xi^2) \Pr(\sigma_{\boldsymbol{\gamma}}) \Pr(\tau_1^2) \Pr(\tau_2^2) \Pr(\omega)
\end{aligned}
$$

For the k-th element in l-block, the conditional distribution of Γ_{lk} comes from a mixture distribution, i.e.,

$$
\Gamma_{lk} | \widehat{\boldsymbol{\Gamma}}_l, \widehat{\boldsymbol{\gamma}}_l, \beta_1, \beta_2, \tau_1^2, \tau_2^2, \xi^2 \sim (1 - \eta_l)\ \mathcal{N}(\widetilde{\mu}_{lk0}, \widetilde{\sigma}_{lk0}^2) + \eta_l\ \mathcal{N}(\widetilde{\mu}_{lk1}, \widetilde{\sigma}_{lk1}^2), \tag{9}
$$

where

$$
\begin{aligned}
-\frac{1}{2\widetilde{\sigma}_{lk0}^2} &= -\frac{1}{2} \left(\frac{1}{1 - \widehat{\rho}_e^2} \frac{\widehat{\mathbf{R}}_{kk}^{(l)}}{\widehat{\mathbf{s}}_{\boldsymbol{\Gamma}_{lk}}^2} + \frac{1}{\xi^2 \tau_1^2} \right) \\
\frac{\widetilde{\mu}_{lk0}}{\widetilde{\sigma}_{lk0}} &= \frac{1}{1 - \widehat{\rho}_e^2} \left\{ \frac{\widehat{\Gamma}_{lk}}{\widehat{\mathbf{s}}_{\boldsymbol{\Gamma}_{lk}}^2} - \sum_{j \neq k} \left(\frac{\widehat{\mathbf{R}}_{jk}^{(l)} \Gamma_{lj}}{\widehat{\mathbf{s}}_{\boldsymbol{\Gamma}_{lj}}} \right) \frac{1}{\widehat{\mathbf{s}}_{\boldsymbol{\Gamma}_{lk}}} - \widehat{\rho}_e \frac{\widehat{\gamma}_{lk}}{\widehat{\mathbf{s}}_{\boldsymbol{\gamma}_{lk}} \widehat{\mathbf{s}}_{\boldsymbol{\Gamma}_{lk}}} + \left(\sum_{i=1}^{p} \frac{\widehat{\mathbf{R}}_{jk}^{(l)} \gamma_{li}}{\widehat{\mathbf{s}}_{\gamma_{li}}} \right) \frac{\widehat{\rho}_e}{\widehat{\mathbf{s}}_{\boldsymbol{\Gamma}_{lk}}} \right\} + \frac{\beta_1 \gamma_{lk}}{\xi^2 \tau_1^2} \\
-\frac{1}{2\widetilde{\sigma}_{lk1}^2} &= -\frac{1}{2} \left(\frac{1}{1 - \widehat{\rho}_e^2} \frac{\widehat{\mathbf{R}}_{kk}^{(l)}}{\widehat{\mathbf{s}}_{\boldsymbol{\Gamma}_{lk}}^2} + \frac{1}{\tau_2^2} \right) \\
\frac{\widetilde{\mu}_{lk1}}{\widetilde{\sigma}_{lk1}} &= \frac{1}{1 - \widehat{\rho}_e^2} \left\{ \frac{\widehat{\Gamma}_{lk}}{\widehat{\mathbf{s}}_{\boldsymbol{\Gamma}_{lk}}^2} - \sum_{j \neq k} \left(\frac{\widehat{\mathbf{R}}_{jk}^{(l)} \Gamma_{lj}}{\widehat{\mathbf{s}}_{\boldsymbol{\Gamma}_{lj}}} \right) \frac{1}{\widehat{\mathbf{s}}_{\boldsymbol{\Gamma}_{lk}}} - \widehat{\rho}_e \frac{\widehat{\gamma}_{lk}}{\widehat{\mathbf{s}}_{\boldsymbol{\gamma}_{lk}} \widehat{\mathbf{s}}_{\boldsymbol{\Gamma}_{lk}}} + \left(\sum_{i=1}^{p} \frac{\widehat{\mathbf{R}}_{jk}^{(l)} \gamma_{li}}{\widehat{\mathbf{s}}_{\gamma_{li}}} \right) \frac{\widehat{\rho}_e}{\widehat{\mathbf{s}}_{\boldsymbol{\Gamma}_{lk}}} \right\} + \frac{\beta_2 \gamma_{lk}}{\tau_2^2}
\end{aligned}
$$

For the k-th element in the l-block, the conditional distribution of γ_{lk} also comes from a mixture of two normal distributions, i.e.,

$$
\gamma_{lk} | \widehat{\boldsymbol{\gamma}}_l, \boldsymbol{\Gamma}_l, \eta_l, \beta_1, \beta_2, \tau_1^2, \tau_2^2, \sigma_{\boldsymbol{\gamma}}^2, \xi^2 \sim (1 - \eta_l)\ \mathcal{N}(\mu_{lk0}, \sigma_{lk0}) + \eta_l\ \mathcal{N}(\mu_{lk1}, \sigma_{lk1}^2), \tag{10}
$$

where

$$
\begin{aligned}
-\frac{1}{2\sigma_{lk0}^2} &= -\frac{1}{2}\left(\frac{1}{1-\widehat{\rho}_e^2}\frac{\widehat{\mathbf{R}}_{kk}^{(l)}}{\widehat{\mathbf{s}}_{\gamma_{lk}}^2}+\frac{\beta_1^2}{\xi^2\tau_1^2}+\frac{1}{\sigma_{\boldsymbol{\gamma}}^2}\right),\\
\frac{\mu_{lk0}}{\sigma_{lk0}^2} &= \frac{1}{1-\widehat{\rho}_e^2}\left\{\frac{\widehat{\gamma}_{lk}}{\widehat{\mathbf{s}}_{\gamma_{lk}}^2}-\sum_{j\neq k}\left(\frac{\widehat{\mathbf{R}}_{jk}^{(l)}\gamma_{lj}}{\widehat{\mathbf{s}}_{\gamma_{lj}}}\right)\frac{1}{\widehat{\mathbf{s}}_{\gamma_{lk}}}-\widehat{\rho}_e\frac{\widehat{\Gamma}_{lk}}{\widehat{\mathbf{s}}_{\gamma_{lk}}\widehat{\mathbf{s}}_{\Gamma_{lk}}}+\left(\sum_{i=1}^{p}\frac{\widehat{\mathbf{R}}_{ik}^{(l)}\Gamma_{li}}{\widehat{\mathbf{s}}_{\Gamma_{li}}}\right)\frac{\widehat{\rho}_e}{\widehat{\mathbf{s}}_{\gamma_{lk}}}\right\}+\frac{\beta_1\Gamma_{lk}}{\xi^2\tau_1^2},\\
-\frac{1}{2\sigma_{lk1}^2} &= -\frac{1}{2}\left(\frac{1}{1-\widehat{\rho}_e^2}\frac{\widehat{\mathbf{R}}_{kk}^{(l)}}{\widehat{\mathbf{s}}_{\gamma_{lk}}^2}+\frac{\beta_2^2}{\tau_2^2}+\frac{1}{\sigma_{\boldsymbol{\gamma}}^2}\right),\\
\frac{\mu_{lk1}}{\sigma_{lk1}^2} &= \frac{1}{1-\widehat{\rho}_e^2}\left\{\frac{\widehat{\gamma}_{lk}}{\widehat{\mathbf{s}}_{\gamma_{lk}}^2}-\sum_{j\neq k}\left(\frac{\widehat{\mathbf{R}}_{jk}^{(l)}\gamma_{lj}}{\widehat{\mathbf{s}}_{\gamma_{lj}}}\right)\frac{1}{\widehat{\mathbf{s}}_{\gamma_{lk}}}-\widehat{\rho}_e\frac{\widehat{\Gamma}_{lk}}{\widehat{\mathbf{s}}_{\gamma_{lk}}\widehat{\mathbf{s}}_{\Gamma_{lk}}}+\left(\sum_{i=1}^{p}\frac{\widehat{\mathbf{R}}_{ik}^{(l)}\Gamma_{li}}{\widehat{\mathbf{s}}_{\Gamma_{li}}}\right)\frac{\widehat{\rho}_e}{\widehat{\mathbf{s}}_{\gamma_{lk}}}\right\}+\frac{\beta_2\Gamma_{lk}}{\tau_2^2}.
\end{aligned}
$$

Conditioning on the data and other parameters, both β_1 and β_2 have normal distributions, i.e.,

$$\beta_1|\boldsymbol{\Gamma},\boldsymbol{\gamma},\boldsymbol{\eta},\tau_1^2,\xi^2 \sim \mathcal{N}(\mu_{\beta_1},\sigma_{\beta_1}^2),\quad \beta_2|\boldsymbol{\Gamma},\boldsymbol{\gamma},\boldsymbol{\eta},\tau_2^2 \sim \mathcal{N}(\mu_{\beta_2},\sigma_{\beta_2}^2), \tag{11}$$

where

$$\left\{\begin{array}{l} -\frac{1}{2\sigma_{\beta_1}^2} = -\frac{1}{2}\frac{\sum_{l=1}^{L}(1-\eta_l)\boldsymbol{\gamma}_l^{\mathrm{T}}\boldsymbol{\gamma}_l}{\xi^2\tau_1^2},\\ \frac{\mu_{\beta_1}}{\sigma_{\beta_1}^2} = \frac{\sum_{l=1}^{L}(1-\eta_l)\boldsymbol{\Gamma}_l^{\mathrm{T}}\boldsymbol{\gamma}_l}{\xi^2\tau_1^2}, \end{array}\right. \quad \text{and} \quad \left\{\begin{array}{l} -\frac{1}{2\sigma_{\beta_2}^2} = -\frac{1}{2}\frac{\sum_{l=1}^{L}\eta_l\boldsymbol{\gamma}_l^{\mathrm{T}}\boldsymbol{\gamma}_l}{\tau_2^2},\\ \frac{\mu_{\beta_1}}{\sigma_{\beta_2}^2} = \frac{\sum_{l=1}^{L}\eta_l\boldsymbol{\Gamma}_l^{\mathrm{T}}\boldsymbol{\gamma}_l}{\tau_2^2}. \end{array}\right.$$

The conditional posterior distributions of $\sigma_{\boldsymbol{\gamma}}^2$, τ_1^2,τ_2^2 and ξ^2 are all inverse-gamma,i.e.,

$$\sigma_{\boldsymbol{\gamma}}^2|\boldsymbol{\gamma},a_{\boldsymbol{\gamma}},b_{\boldsymbol{\gamma}} \sim \mathcal{IG}(\widetilde{a}_{\boldsymbol{\gamma}},\widetilde{b}_{\boldsymbol{\gamma}}),\qquad \tau_1^2|\boldsymbol{\Gamma},\boldsymbol{\gamma},\xi^2,a_{\tau1},b_{\tau1} \sim \mathcal{IG}(\widetilde{a}_{\tau1},\widetilde{b}_{\tau1}), \tag{12}$$

$$\tau_2^2|\boldsymbol{\Gamma},\boldsymbol{\gamma},a_{\tau2},b_{\tau2} \sim \mathcal{IG}(\widetilde{a}_{\tau2},\widetilde{b}_{\tau2}),\quad \xi^2|\boldsymbol{\Gamma},\boldsymbol{\gamma},\beta_1,\boldsymbol{\eta} \sim \mathcal{IG}(a_\xi,b_\xi), \tag{13}$$

where $\widetilde{a}_{\boldsymbol{\gamma}} = a_{\boldsymbol{\gamma}}+\frac{p}{2}$, $\widetilde{b}_{\boldsymbol{\gamma}} = b_{\boldsymbol{\gamma}}+\frac{\sum_{l=1}^{L}\boldsymbol{\gamma}_l^{\mathrm{T}}\boldsymbol{\gamma}_l}{2}$, $\widetilde{a}_{\tau1} = a_{\tau1}+\frac{\sum_{l=1}^{L}(1-\eta_l)p_l}{2}$, $\widetilde{b}_{\tau1} = b_{\tau1}+\frac{\sum_{l=1}^{L}(1-\eta_l)(\boldsymbol{\Gamma}_l-\beta_1\boldsymbol{\gamma}_l)^{\mathrm{T}}(\boldsymbol{\Gamma}_l-\beta_1\boldsymbol{\gamma}_l)}{2\xi^2}$, $\widetilde{a}_{\tau2} = a_{\tau2}+\frac{\sum_{l=1}^{L}\eta_l p_l}{2}$, $\widetilde{b}_{\tau2} = b_{\tau2}+\frac{\sum_{l=1}^{L}\eta_l(\boldsymbol{\Gamma}_l-\beta_2\boldsymbol{\gamma}_l)^{\mathrm{T}}(\boldsymbol{\Gamma}_l-\beta_2\boldsymbol{\gamma}_l)}{2}$, $a_\xi = \frac{1}{2}\sum_{l=1}^{L}(1-\eta_l)p_l$ and $b_\xi = \frac{1}{2\tau_1^2}\sum_{l=1}^{L}(1-\eta_l)(\boldsymbol{\Gamma}_l-\beta_1\boldsymbol{\gamma}_l)^{\mathrm{T}}(\boldsymbol{\Gamma}_l-\beta_1\boldsymbol{\gamma}_l)$.

The conditional posterior of ω is also a Beta distribution:

$$\omega|\boldsymbol{\eta}, a, b \sim \text{Beta}\left(a + \sum_{l=1}^{L} \eta_l, b + \sum_{l=1}^{L} (1 - \eta_l)\right). \tag{14}$$

The conditional probability of $\boldsymbol{\eta}$ given $\boldsymbol{\Gamma}$ can be found using Bayes' theorem

$$\begin{aligned} \Pr(\eta_l = 1|\Gamma_k) &= \frac{\Pr(\eta_l = 1)\Pr(\boldsymbol{\Gamma}_l|\eta_l = 1)}{\Pr(\eta_l = 0)\Pr(\boldsymbol{\Gamma}_l|\eta_l = 0) + \Pr(\eta_l = 1)\Pr(\boldsymbol{\Gamma}_l|\eta_l = 1)} \\ &= \frac{\omega \prod_{k=1}^{p_l} \mathcal{N}(\beta_2\gamma_{kl}, \tau_2^2)}{\omega \prod_{k=1}^{p_l} \mathcal{N}(\beta_2\gamma_{kl}, \tau_2^2) + (1-\omega)\prod_{k=1}^{p_l} \mathcal{N}(\beta_1\gamma_{kl}, \xi^2\tau_1^2)}. \end{aligned} \tag{15}$$

Algorithm 2 The parallel Gibbs sampler algorithm for correlated SNPs

Initialize:

repeat
    for $l = 1$ to L **do** — Working Parallel (lines from this **for** to its **end for**)
        for $k = 1$ to p_l **do**
            Sample Γ_{lk} using (9).
        end for
        for $k = 1$ to p_l **do**
            Sample γ_{lk} using (10).
        end for
        Sample η_l using (15).
    end for
    Sample β_1 and β_2 using (11).
    Sample σ_γ^2 , τ_1^2, τ_2^2 and ξ^2 using (12).
    Sample ω using (14).
until Maximum iteration reached

Supplementary Note 2
Pseudo-code

In this section, we present the pseudo-code for the algorithms using correlated SNPs. Comment lines are preceded by % %.

$\mathbf{a}[i]$ denotes the i-th element of a vector $\mathbf{a}$. $\mathbf{a}[s:e]$ denotes a subvector of $\mathbf{a}$, spanning from the s-th to the e-th element. $\mathbf{A}[,i]$ denotes the i-th column of a matrix $\mathbf{A}$. $\mathbf{a} \circ \mathbf{b}$ denotes element-wise multiplication. $\mathbf{a}/\mathbf{b}$ denotes element-wise division. In addition, we denote the

diagonal of a matrix $\mathbf{A}$ by diag($\mathbf{A}$). A square diagonal matrix with the elements of vector $\mathbf{a}$ on the main diagonal is denoted by diagmat($\mathbf{a}$).

% % Input observations and prior parameters.

Input: $\widehat{\boldsymbol{\gamma}}$, $\widehat{\boldsymbol{\Gamma}}$, $\widehat{\mathbf{s}}^2_{\boldsymbol{\gamma}}$, $\widehat{\mathbf{s}}^2_{\boldsymbol{\Gamma}}, \widehat{\rho}_e, \widehat{\mathbf{R}}, \mathbf{G}; a_{\boldsymbol{\gamma}} \leftarrow 0, b_{\boldsymbol{\gamma}} \leftarrow 0, a_{\tau 1} \leftarrow 0, b_{\tau 1} \leftarrow 0, a_{\tau 2} \leftarrow 0, b_{\tau 2} \leftarrow 0, a \leftarrow 2, b \leftarrow L$.

% % $\mathbf{G}$ is a $L \times 2$ matrix, which stores the group information, start with the first element and end with the second element of each row.

Output: β_1, β_2, $\boldsymbol{\eta}$.

% % Initialize latent variables.

$\omega \leftarrow 0.1, \beta_1 \leftarrow 0.01, \beta_2 \leftarrow 0.01, \sigma^2_{\boldsymbol{\gamma}} \leftarrow 0.01, \tau_1^2 \leftarrow 0.01, \tau_2^2 \leftarrow 0.01, \xi^2 \leftarrow 0.01,\ \boldsymbol{\gamma} = 0.01 * \mathbf{1_p}, \boldsymbol{\Gamma} = \mathbf{0.01} * \mathbf{1_p}, \boldsymbol{\eta} = \mathbf{0_L}$.

$\boldsymbol{\gamma}_{\eta^0} \leftarrow \mathbf{0_L}; \boldsymbol{\gamma}_{\eta^1} \leftarrow \mathbf{0_L}; \boldsymbol{\gamma}_{\boldsymbol{\Gamma}\eta^0} \leftarrow \mathbf{0_L}; \boldsymbol{\gamma}_{\boldsymbol{\Gamma}\eta^1} \leftarrow \mathbf{0_L}; \eta_\mathbf{0}^\mathbf{L} \leftarrow \mathbf{0_L}\eta_\mathbf{1}^\mathbf{L} \leftarrow \mathbf{0_L}; \mathbf{t}_{\tau_\mathbf{1}^\mathbf{2}} \leftarrow \mathbf{0_L}; \mathbf{t}_{\tau_\mathbf{2}^\mathbf{2}} \leftarrow \mathbf{0_L}$;

$\rho_1^* \leftarrow 1/(1 - \widehat{\rho}_e * \widehat{\rho}_e)$; $\rho_2^* \leftarrow \widehat{\rho}_e/(1 - \widehat{\rho}_e * \widehat{\rho}_e)$; $\rho_{\gamma\Gamma} \leftarrow \rho_1^* \widehat{\mathbf{s}}_{\boldsymbol{\gamma}l}/\widehat{\mathbf{s}}_{\boldsymbol{\Gamma}}$; $\rho_{\Gamma\gamma} \leftarrow \rho_1^* \widehat{\mathbf{s}}_{\boldsymbol{\Gamma}l}/\widehat{\mathbf{s}}_{\boldsymbol{\gamma}}$;

$\hat{\zeta}_\gamma \leftarrow \rho_2^* \cdot \widehat{\boldsymbol{\gamma}}_l/(\widehat{\mathbf{s}}_{\boldsymbol{\Gamma}l} \circ \widehat{\mathbf{s}}_{\boldsymbol{\gamma}})$; $\hat{\zeta}_\Gamma \leftarrow \rho_2^* \cdot \widehat{\boldsymbol{\Gamma}}_l/(\widehat{\mathbf{s}}_{\boldsymbol{\Gamma}l} \circ \widehat{\mathbf{s}}_{\boldsymbol{\gamma}})$;

for $i = 1$ to number of MCMC iterations **do**

$logw \leftarrow \log(\omega/(1-\omega))$

for $l = 1 \in 1, \cdots, L$ **do**

% % Parameters for updating $\boldsymbol{\Gamma}_l$.

$s_l \leftarrow \mathbf{G}[l,1]; e_l \leftarrow \mathbf{G}[l,2]$; $\widehat{\mathbf{R}}^{(l)} \leftarrow \widehat{\mathbf{R}}[s_l : e_l, s_l : e_l]$; $\mathbf{d}_l = \text{diag}(\widehat{\mathbf{R}}^{(l)})$;

$\widehat{\mathbf{s}}^2_{\boldsymbol{\gamma}l} \leftarrow \widehat{\mathbf{s}}^2_{\boldsymbol{\gamma}}[s_l : e_l]$; $\widehat{\mathbf{s}}^2_{\boldsymbol{\Gamma}l} \leftarrow \widehat{\mathbf{s}}^2_{\boldsymbol{\Gamma}}[s_l : e_l]$; $\widehat{\boldsymbol{\gamma}}_l \leftarrow \widehat{\boldsymbol{\gamma}}[s_l : e_l]$; $\widehat{\boldsymbol{\Gamma}}_l \leftarrow \widehat{\boldsymbol{\Gamma}}[s_l : e_l]$;

$\hat{\zeta}_{\gamma l} \leftarrow \hat{\zeta}_\gamma[s_l : e_l]$; $\hat{\zeta}_{\Gamma l} \leftarrow \hat{\zeta}_\Gamma[s_l : e_l]$;

$\rho^l_{\gamma\Gamma} \leftarrow \rho_{\gamma\Gamma}[s_l : e_l]$; $\rho^l_{\gamma\Gamma} \leftarrow \rho_{\Gamma\gamma}[s_l : e_l]$;

$\boldsymbol{\gamma}_l \leftarrow \boldsymbol{\gamma}[s_l : e_l]$; $\boldsymbol{\Gamma}_l \leftarrow \boldsymbol{\Gamma}[s_l : e_l]; p_l \leftarrow \text{length}(\boldsymbol{\gamma}_l)$;

$\delta_{\boldsymbol{\Gamma}l} \leftarrow \text{diag}(\rho_1 \widehat{\mathbf{R}}^{(l)} * \text{diagmat}(1/\widehat{\mathbf{s}}_{\boldsymbol{\Gamma}})); \delta_{\boldsymbol{\gamma}l} \leftarrow \text{diag}(\rho_1 \widehat{\mathbf{R}}^{(l)} * \text{diagmat}(1/\widehat{\mathbf{s}}_{\boldsymbol{\gamma}}))$;

$\Delta_{\boldsymbol{\Gamma}l} \leftarrow \rho_1^* \widehat{\mathbf{R}}^{(l)} * \text{diagmat}(1/\widehat{\mathbf{s}}_{\boldsymbol{\Gamma}l}); \Delta_{\boldsymbol{\gamma}l} \leftarrow \rho_1^* \widehat{\mathbf{R}}^{(l)} * \text{diagmat}(1/\widehat{\mathbf{s}}_{\boldsymbol{\gamma}l})$;

$v^2_{\mathbf{\Gamma}1} \leftarrow 1/(\rho_2^* \widehat{\mathbf{\Gamma}}_l/(\widehat{\mathbf{s}}_{\gamma l} \circ \widehat{\mathbf{s}}_{\mathbf{\Gamma} l}) + 1/(\tau_1^2 \xi^2))$; $v^2_{\mathbf{\Gamma}2} \leftarrow 1/(\rho_2^* \widehat{\mathbf{\Gamma}}_l/(\widehat{\mathbf{s}}_{\gamma l} \circ \widehat{\mathbf{s}}_{\mathbf{\Gamma} l}) + 1/\tau_2^2)$;

$v^2_{\boldsymbol{\gamma}1} \leftarrow 1/(\rho_2^* \widehat{\gamma}_l/(\widehat{\mathbf{s}}_{\boldsymbol{\gamma} l} \circ \widehat{\mathbf{s}}_{\mathbf{\Gamma} l}) + \beta_1 * \beta_1/(\tau_1^2 \xi^2) + 1/\sigma_\gamma^2)$; $v^2_{\boldsymbol{\gamma}2} \leftarrow 1/(\rho_2^* \widehat{\gamma}_l/(\widehat{\mathbf{s}}_{\boldsymbol{\gamma} l} \circ \widehat{\mathbf{s}}_{\mathbf{\Gamma} l}) + \beta_2 * \beta_2/\tau_2^2 + 1/\sigma_\gamma^2)$;

$\mu_{\mathbf{\Gamma} l} \leftarrow \rho_1^* \widehat{\mathbf{R}}^{(l)} * \text{diagmat}(1/\mathbf{s}_{\mathbf{\Gamma} l}) * \mathbf{\Gamma}_l; \mu_{\boldsymbol{\gamma} l} \leftarrow \rho_1^* \widehat{\mathbf{R}}^{(l)} * \text{diagmat}(1/\mathbf{s}_{\boldsymbol{\gamma} l}) * \boldsymbol{\gamma}_l$;

% % Sample $\mathbf{\Gamma}_l$ in group l.

if $\eta[l] = 1$ **then**

for $k = 1 \in 1, \cdots, p_l$ **do**

$t_1 \leftarrow \mu_{\mathbf{\Gamma} l} - \mathbf{\Gamma}_l[k] \cdot \Delta_{\mathbf{\Gamma} l}[, k]$;

$\mu^k_{\mathbf{\Gamma} l} \leftarrow \mu_{\mathbf{\Gamma} l}[k] - \delta_{\mathbf{\Gamma} l}[k] \cdot \mathbf{\Gamma}_l[k]$;

$\widetilde{m}_{l1} \leftarrow (\rho^l_{\Gamma\gamma}[k] - \mu^k_{\mathbf{\Gamma} l}/\widehat{\mathbf{\Gamma}}_l[k] - \hat{\zeta}_{\boldsymbol{\gamma} l}[k] + \rho \cdot \mu_{\gamma l}[k]/\widehat{\mathbf{\Gamma}}_l[k] + \beta_2 \cdot \Gamma_l[k]/\tau_2^2))/v^2_{\Gamma 2}[k]$;

% % Draw $\mathbf{\Gamma}_l[k]$ from normal distribution.

$\mathbf{\Gamma}_l[k] \sim \mathcal{N}(\widetilde{m}_{l1}, v^2_{\Gamma 1}[k])$;

$\mu_{\mathbf{\Gamma} l} \leftarrow t_1 + \mathbf{\Gamma}_l[k] \cdot \Delta_{\mathbf{\Gamma} l}[, k]$;

end for

else

for $k = 1 \in 1, \cdots, p_l$ **do**

$t_0 \leftarrow \mu_{\mathbf{\Gamma} l} - \mathbf{\Gamma}_l[k] \cdot \Delta_{\mathbf{\Gamma} l}[, k]$;

$\mu^k_{\mathbf{\Gamma} l} \leftarrow \mu_{\mathbf{\Gamma} l}[k] - \delta_{\mathbf{\Gamma} l}[k] \cdot \mathbf{\Gamma}_l[k]$;

$\widetilde{m}_{l0} \leftarrow (\rho^l_{\Gamma\gamma}[k] - \mu^k_{\mathbf{\Gamma} l}/\widehat{\mathbf{\Gamma}}_l[k] - \hat{\zeta}_{\boldsymbol{\gamma} l}[k] + \rho \cdot \mu_{\gamma l}[k]/\widehat{\mathbf{\Gamma}}_l[k] + \beta_1 \cdot \Gamma_l[k]/(\tau_1^2 \xi^2))/v^2_{\Gamma 1}[k]$;

% % Draw $\mathbf{\Gamma}_l[k]$ from normal distribution.

$\mathbf{\Gamma}_l[k] \sim \mathcal{N}(\widetilde{m}_{l0}, v^2_{\Gamma 0}[k])$;

$\mu_{\mathbf{\Gamma} l} \leftarrow t_0 + \mathbf{\Gamma}_l[k] \cdot \Delta_{\mathbf{\Gamma} l}[, k]$;

end for

end if

% % Sample $\boldsymbol{\gamma}_l$ in group l.

if $\eta[l] = 1$ **then**

for $k = 1 \in 1, \cdots, p_l$ **do**

$t_1 \leftarrow \mu_{\boldsymbol{\gamma} l} - \boldsymbol{\gamma}_l[k] \cdot \Delta_{\boldsymbol{\gamma} l}[,k];$

$\mu^k_{\boldsymbol{\gamma} l} \leftarrow \mu_{\boldsymbol{\gamma} l}[k] - \delta_{\boldsymbol{\gamma} l}[k] \cdot \boldsymbol{\gamma}_l[k];$

$m_{l1} \leftarrow (\rho^l_{\gamma\Gamma}[k] - \mu^k_{\boldsymbol{\gamma} l}/\widehat{\boldsymbol{\gamma}}_l[k] - \hat{\zeta}_{\boldsymbol{\Gamma} l}[k] + \rho \cdot \mu_{\Gamma l}[k]/\widehat{\boldsymbol{\gamma}}_l[k] + \beta_2 \cdot \gamma_l[k]/\tau_2^2))/v^2_{\gamma 2}[k];$

% % Draw $\boldsymbol{\gamma}_l[k]$ from normal distribution.

$\boldsymbol{\gamma}_l[k] \sim \mathcal{N}(m_{l1}, v^2_{\gamma 2}[k]);$

$\mu_{\boldsymbol{\gamma} l} \leftarrow t_1 + \boldsymbol{\gamma}_l[k] \cdot \Delta_{\boldsymbol{\gamma} l}[,k];$

end for

else

for $k = 1 \in 1, \cdots, p_l$ **do**

$t_0 \leftarrow \mu_{\boldsymbol{\gamma} l} - \boldsymbol{\gamma}_l[k] \cdot \Delta_{\boldsymbol{\gamma} l}[,k];$

$\mu^k_{\boldsymbol{\gamma} l} \leftarrow \mu_{\boldsymbol{\gamma} l}[k] - \delta_{\boldsymbol{\gamma} l}[k] \cdot \boldsymbol{\gamma}_l[k];$

$m_{l0} \leftarrow (\rho^l_{\gamma\Gamma}[k] - \mu^k_{\boldsymbol{\gamma} l}/\widehat{\boldsymbol{\gamma}}_l[k] - \hat{\zeta}_{\boldsymbol{\Gamma} l}[k] + \rho \cdot \mu_{\Gamma l}[k]/\widehat{\boldsymbol{\gamma}}_l[k] + \beta_1 \cdot \gamma_l[k]/(\tau_1^2 \xi^2)))/v^2_{\gamma 1}[k];$

% % Draw $\boldsymbol{\gamma}_l[k]$ from normal distribution.

$\boldsymbol{\gamma}_l[k] \sim \mathcal{N}(m_{l0}, v^2_{\gamma 1}[k]);$

$\mu_{\boldsymbol{\gamma} l} \leftarrow t_0 + \boldsymbol{\gamma}_l[k] \cdot \Delta_{\boldsymbol{\gamma} l}[,k];$

end for

end if

% Update $\eta[l]$.

$\epsilon_2 \leftarrow \boldsymbol{\Gamma}_l - \beta_2 \boldsymbol{\gamma}_l;\ \ \epsilon_1 \leftarrow \boldsymbol{\Gamma}_l - \beta_1 \boldsymbol{\gamma}_l;$

$t_2 \leftarrow logw - sum(\epsilon_2 \circ \epsilon_2)/(2\tau_2^2) - p_l \cdot \log(\tau_2^2)/2 + sum(\epsilon_1 \circ \epsilon_1)/(2\tau_1^2\xi^2) + p_1 \cdot \log(\tau_1^2\xi^2)/2.$

$prob \leftarrow 1/(1 + \exp(-t_2));$

$\eta[l] \sim \text{Binom}(prob);$

% Renew some terms for the following procedure.

$\boldsymbol{\gamma}_{\eta^0}[s_l : e_l] \leftarrow (1-\eta[l]) \cdot sum(\boldsymbol{\gamma}_l \circ \boldsymbol{\gamma}_l);$

$\boldsymbol{\gamma}_{\eta^1}[s_l : e_l] \leftarrow \eta[l] \cdot sum(\boldsymbol{\gamma}_l \circ \boldsymbol{\gamma}_l);$

$\boldsymbol{\gamma}_{\boldsymbol{\Gamma}\eta^0}[s_l : e_l] \leftarrow (1-\eta[l]) \cdot sum(\boldsymbol{\Gamma}_l \circ \boldsymbol{\gamma}_l);$

$\boldsymbol{\gamma}_{\boldsymbol{\Gamma}\eta^1}[s_l : e_l] \leftarrow \eta[l] \cdot sum(\boldsymbol{\Gamma}_l \circ \boldsymbol{\gamma}_l);$

$\eta_0^L[s_l : e_l] \leftarrow p_l \cdot (1-\eta[l]);$

$\eta_1^L[s_l : e_l] \leftarrow p_l \cdot \eta[l];$

$t_{\tau_1^2}[s_l : e_l] \leftarrow (1-\eta[l]) \cdot sum((\boldsymbol{\Gamma}_l - \beta_1 \cdot \boldsymbol{\gamma}_l) \circ (\boldsymbol{\Gamma}_l - \beta_1 \cdot \boldsymbol{\gamma}_l));$

$t_{\tau_2^2}[s_l : e_l] \leftarrow \eta[l] \cdot sum((\boldsymbol{\Gamma}_l - \beta_2 \cdot \boldsymbol{\gamma}_l) \circ (\boldsymbol{\Gamma}_l - \beta_2 \cdot \boldsymbol{\gamma}_l));$

end for

% % Draw β_1 from normal distribution.

if sum(η=p) **then**

$\beta_1 \leftarrow 0.$

else

$\sigma_{\beta 1}^2 \leftarrow \tau_1^2 \xi^2 / sum(\boldsymbol{\gamma}_{\eta^0})$

$m_{\beta 1} \leftarrow \sigma_{\beta 1}^2 sum(\boldsymbol{\gamma}_{\boldsymbol{\Gamma}\eta^0}) / (\tau_1^2 \xi^2)$

$\beta_1 \sim \mathcal{N}(m_{\beta 1}, \sigma_{\beta 1}^2).$

end if

% % Draw β_2 from normal distribution.

if sum(η=0) **then**

$\beta_2 \leftarrow 0;$

else

$\sigma_{\beta 2}^2 \leftarrow \tau_2^2 / sum(\boldsymbol{\gamma}_{\eta^1})$

$m_{\beta 2} \leftarrow \sigma_{\beta 2}^2 sum(\boldsymbol{\gamma}_{\boldsymbol{\Gamma}\eta^1}) / \tau_2^2$

$\beta_2 \sim \mathcal{N}(m_{\beta 2}, \sigma_{\beta 2}^2).$

end if

$\widetilde{a}_{\gamma} \leftarrow a_{\gamma} + p/2$; $\widetilde{b}_{\gamma} \leftarrow b_{\gamma} + sum(\boldsymbol{\gamma} \circ \boldsymbol{\gamma})/2$.

$\widetilde{a}_{\tau 1} \leftarrow a_{\tau 1} + sum(\eta_0^L)/2$; $\widetilde{b}_{\tau 1} \leftarrow b_{\tau 1} + sum(t_{\tau_1^2})/(2\xi^2)$.

$\widetilde{a}_{\tau 2} \leftarrow a_{\tau 2} + sum(\eta_1^L)/2$; $\widetilde{b}_{\tau_2} \leftarrow b_{\tau_2} + sum(t_{\tau_2^2})/(2)$.

$a_{\xi} \leftarrow sum(\eta_0^L)/2$; $b_{\xi} \leftarrow sum(t_{\tau_1^2})/(2\tau_1^2)$.

% % Draw $\sigma_{\boldsymbol{\gamma}}^2$ from Inverse-gamma distribution.

$\sigma_{\boldsymbol{\gamma}}^2 \sim \mathcal{IG}(\widetilde{a}_{\gamma}, \widetilde{b}_{\gamma})$.

% % Draw τ_1^2 from Inverse-gamma distribution.

$\tau_1^2 \sim \mathcal{IG}(\widetilde{a}_{\tau 1}, \widetilde{b}_{\tau 1})$.

% % Draw τ_2^2 from Inverse-gamma distribution.

$\tau_2^2 \sim \mathcal{IG}(\widetilde{a}_{\tau 2}, \widetilde{b}_{\tau 2})$.

% % Draw ξ^2 from Inverse-gamma distribution.

$\xi^2 \sim \mathcal{IG}(a_{\xi}, b_{\xi})$.

% % Draw ω from Beta distribution.

$\omega \sim \mathrm{Beta}(a + sum(\boldsymbol{\eta}), b + sum(\mathbf{1} - \boldsymbol{\eta}))$.

% % Store η of each iteration into a matrix $\mathbf{H}$.

$\mathbf{H}.col(i) \leftarrow \eta$

end for

Supplementary Note 3
Additional simulation results

3.1 The identifiability of causal effect

In this subsection, we discuss and evaluate the identifiability of causal effect. Following existing literature [1, 2], our model also assumes that all IVs could have potential uncorrelated pleiotropic effect while only a sparse proportion of IVs have correlated pleiotropic effect. As a consequence of the assumption, the variability of Γ_k is larger for the β_2 group of SNPs

than the β_1 group because of the existence of $\widetilde{\alpha}_k$.

In Equation (7) of the main text, we have

$$\Gamma_k|\beta_1, \beta_2, \gamma_k, \eta_k, \tau_1^2, \tau_2^2 \sim \begin{cases} \mathcal{N}(\beta_1\gamma_k, \tau_1^2), & \eta_k = 0 \\ \mathcal{N}(\beta_2\gamma_k, \tau_2^2), & \eta_k = 1 \end{cases}, \tag{16}$$

where $\tau_1^2 = \sigma_\theta^2$ for IVs with potential UHP only and $\tau_2^2 = \sigma_\theta^2 + \sigma_\alpha^2$ with both potential UHP and CHP. In this formulation, $\tau_2^2 > \tau_1^2$. Since both τ_1^2 and τ_2^2 are model parameters, we can obtain their estimates using MCMC.

To evaluate the validity of the condition in different settings, we conducted the following simulations. For each block of IV sets l, we reparameterize γ_{lk} and α_{lk} as follows

$$\begin{aligned} \gamma_{lk} &\sim \mathcal{N}\left(0, \sigma_\gamma^2\right) \\ \alpha_{lk} &= \delta \cdot \gamma_{lk} + \widetilde{\alpha}_{lk} \end{aligned}$$

where $\widetilde{\alpha}_{lk} \sim \mathcal{N}\left(0, \sigma_\alpha^2\right)$ and $\gamma_{lk} \perp\!\!\!\perp \widetilde{\alpha}_{lk}$. Thus, for IVs with no CHP effect, the causal effect from exposure to outcome is given by β_1. For IVs with both UHP and CHP effects, the causal effect is $\beta_2 = \delta + \beta_1$, and δ is the difference/bias in estimation due to CHP. To assess the identifiability of causal effects, we vary the value of δ and conduct the simulations with 100 replications. We examine the proportion of sets of estimates with $\hat{\tau}_1^2 < \hat{\tau}_2^2$. We set the true causal effect equals to 1. In Table 1 , we show that when the sparsity levels of IVs with CHP is sparse (0.1) or moderately sparse (0.3), using the condition $\hat{\tau}_1^2 < \hat{\tau}_2^2$ to identify the causal effect $\hat{\beta}_1$ works well. However, when the proportion of IVs with CHP becomes non-sparse (0.5) and when the difference between β_1 and β_2 (i.e., δ) is large (1 or -1), the identification has some issues and the estimated β_1 start to show biases. It should be noted that when the proportion of IVs with CHP is non-sparse, CHP and UHP effects becomes statistically unidentifiable. Thus, in existing literature, it is commonly assumed that the

δ	Proportions of IV with CHP: 0.1			Proportions of IV with CHP: 0.3			Proportions of IV with CHP: 0.5		
	$\hat{\beta}_1$	$\hat{\beta}_2$	Prop. of replications with $\hat{\tau}_1^2 < \hat{\tau}_2^2$ (%)	$\hat{\beta}_1$	$\hat{\beta}_2$	Prop. of replications with $\hat{\tau}_1^2 < \hat{\tau}_2^2$ (%)	$\hat{\beta}_1$	$\hat{\beta}_2$	Prop. of replications with $\hat{\tau}_1^2 < \hat{\tau}_2^2$ (%)
0.0010	1.004	1.007	100	0.996	1.001	100	1.006	0.491	71
0.1000	0.997	1.111	100	1.003	1.083	99	1.020	0.513	73
1.0000	0.998	1.726	100	1.046	1.498	100	1.148	0.657	55
-1.0000	0.992	0.299	100	0.952	0.482	100	0.872	0.161	72

Supplementary Table 1: An evaluation of the identifiability of causal effects in various simulation settings, using the condition $\hat{\tau}_1^2 < \hat{\tau}_2^2$. Here $\sigma_\alpha^2 = 0.1, h_\gamma^2 = 0.1, h_\alpha^2 = 0.05, h_\theta^2 = 0.05, r = 0.4, p = 1,000$.

proportion of IVs with CHP is sparse or moderately sparse [3, 4, 1].

As a conclusion, when the proportion of IVs with CHP effect is sparse or moderately sparse (< 0.5), the condition of $\hat{\tau}_1^2 < \hat{\tau}_2^2$ works very well in identifying the true causal effects. When the proportion is non-sparse (0.5) and the confounding bias (δ) is large, the identification condition may lead to biased estimation.

3.2 Simulation results for different p and $\rho_{\alpha\gamma}$

In this subsection, we provide additional results on type I error rate with different number of IVs ($p = 2,000$) and a stronger correlation between IV-to-exposure and CHP effects ($\rho_{\alpha\gamma} = 0.8$), see Fig. 1. To compare the bias of causal effect estimate derived from each method, we show the box plot of point estimates for each method in Fig. 2, with the true causal effect $\beta_1 = 1$. We further summarize the mean bias of causal effect estimates from MR-CUE and other methods in Table 2. The mean bias of MR-CUE is the smallest among all methods. Also, We compare the power of each method by varying $h_\gamma^2 \in \{0, 10^{-3.5}, 10^{-3}, 10^{-2.5}, 10^{-2}, 10^{-1.5}, 0.1\}$ with different setting, i.e., $h_\theta^2 \in \{0.02, 0.05\}$, $h_\alpha \in \{0.05, 0.1\}$, $r \in \{0.4, 0.8\}$ and $p \in \{1000, 2000\}$. One can found the corresponding results in Fig. 3-6. In addition, we report the false discovery rate (FDR) with $\beta_1 = 0$ and $\beta_1 = 0.1$, respectively. The corresponding result can be found in Fig. 7. We evaluate the performance of identification of IV with CHP ($\boldsymbol{\eta} = 1$) using the area under the receiver operating characteristic (ROC) curve (AUC), see

Fig. 8.

Method	$h_\theta^2 = 0.02, h_\alpha^2 = 0.05$	$h_\theta^2 = 0.05, h_\alpha^2 = 0.1$
MR-CUE	0.071	-0.149
GRAPPLE	-0.207	-0.780
MRMix	1.940	5.470
cML-MA-BIC-DP	2.667	7.035
RAPS	4.446	6.243
MR-Clust	-5.251	-7.092
MR-LDP	-9.620	-30.535
IVW	-11.301	-8.271
MR-Egger	-11.708	-9.026
CAUSE	-24.063	-22.107

Supplementary Table 2: Mean biases of causal effect estimates over 100 replications for competing methods, with $h_\gamma^2 = 0.1, r = 0.4, \rho_{\alpha\gamma} = 0.2, p = 2,000$, all in %.

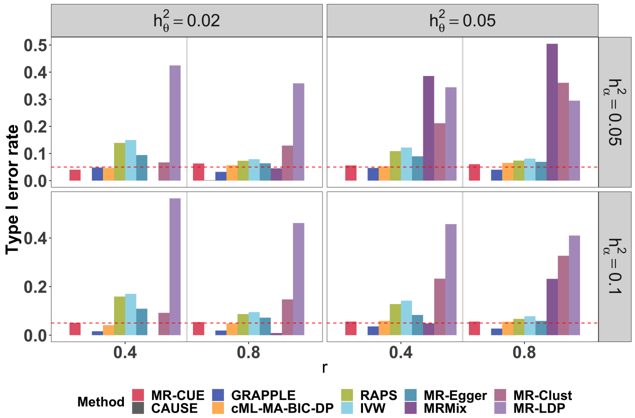

(a) $p = 2,000, \rho_{\alpha\gamma} = 0.2$

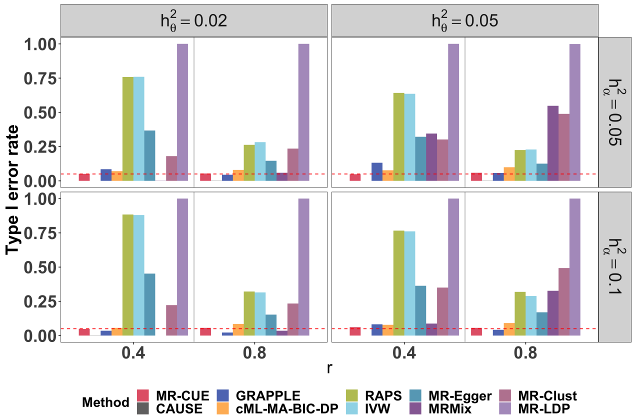

(b) $p = 1,000, \rho_{\alpha\gamma} = 0.8$

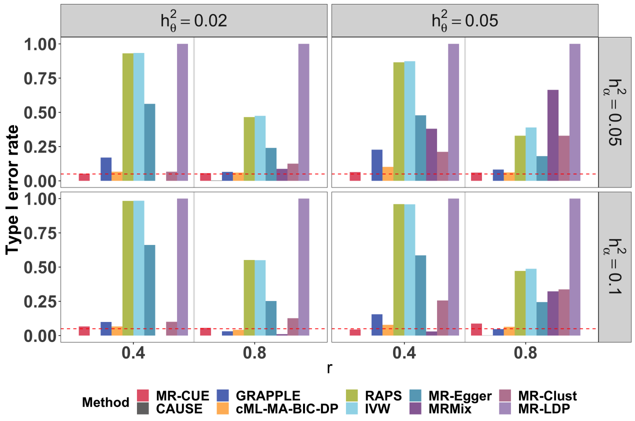

(c) $p = 2,000, \rho_{\alpha\gamma} = 0.8$

Supplementary Figure 1: Evaluation of type I error rate with $\beta_1 = 0$. The number of replicates is 1,000. Note that we evaluate the type I error rate for CAUSE, GRAPPLE, cML-MA-BIC-DP, RAPS, IVW, MR-Egger, MRMix and MR-Clust using independent SNPs.

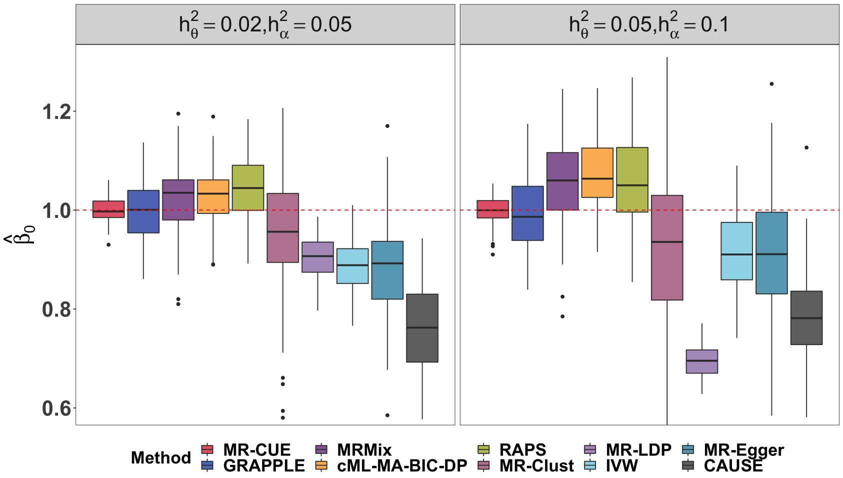

Supplementary Figure 2: The boxplots of point estimates over 100 replications for competing methods, with $h_\gamma^2 = 0.1, r = 0.4, \rho_{\alpha\gamma} = 0.2, p = 2,000$ and sample sizes $n_x = 50,000, n_y = 50,000$. The bounds of the boxes represent 25% and 75% quantiles and the center lines the median values. The top and bottom whiskers are set to the highest/lowest value of the dataset that are included in the 1.5×the interquartile range, outliers are defined as data points that are located outside the whiskers.

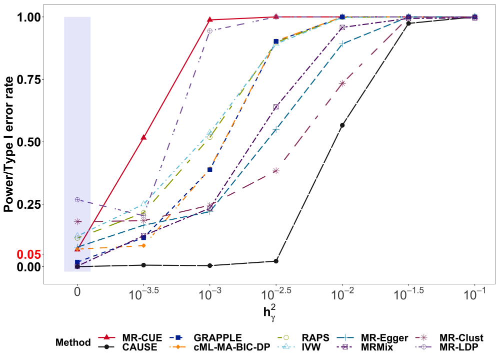

(a) $h^2_\theta = 0.02, h^2_\alpha = 0.05$

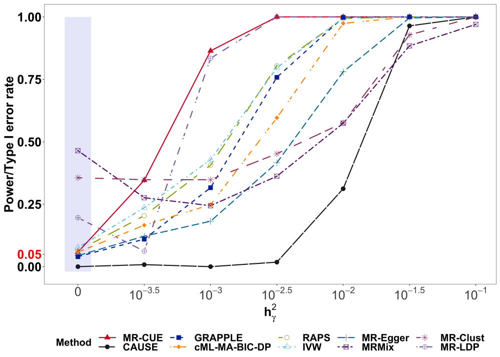

(b) $h^2_\theta = 0.05, h^2_\alpha = 0.05$

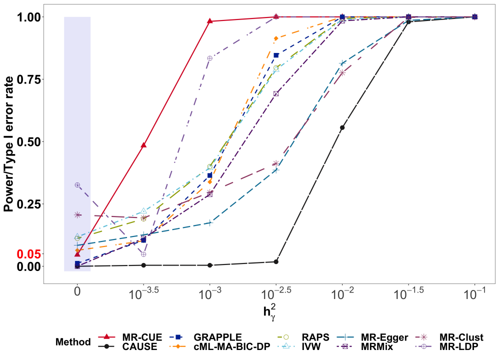

(c) $h^2_\theta = 0.02, h^2_\alpha = 0.1$

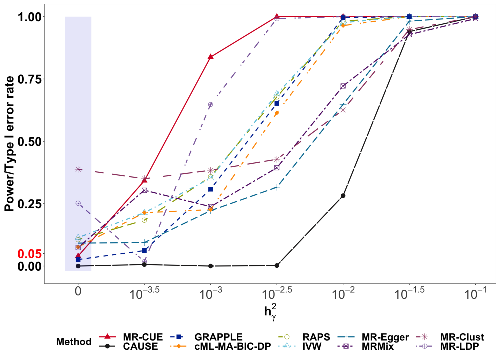

(d) $h^2_\theta = 0.05, h^2_\alpha = 0.1$

Supplementary Figure 3: Power comparison with $\rho_{\alpha\gamma} = 0.2, r = 0.4$ and $p = 1,000$. The number of replicates is 500. Note that we evaluate the type I error rate for CAUSE, GRAPPLE, cML-MA-BIC-DP, RAPS, IVW, MR-Egger, MRMix and MR-Clust using independent SNPs.

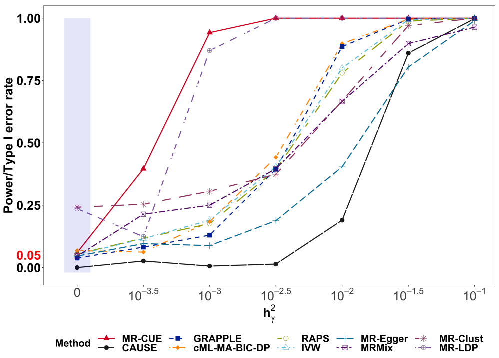

(a) $h^2_\theta = 0.02, h^2_\alpha = 0.05$

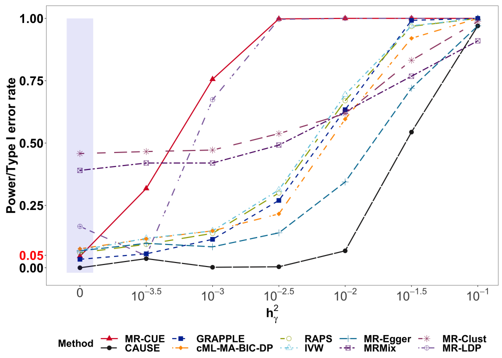

(b) $h^2_\theta = 0.05, h^2_\alpha = 0.05$

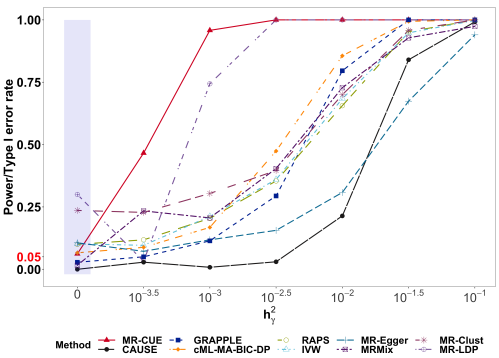

(c) $h^2_\theta = 0.02, h^2_\alpha = 0.1$

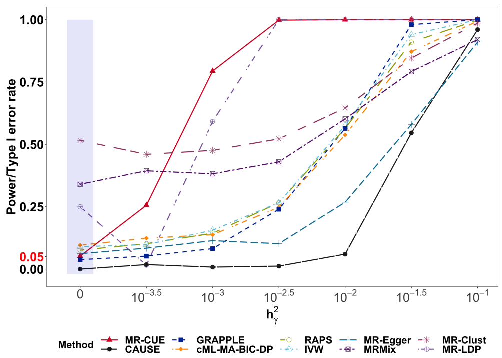

(d) $h^2_\theta = 0.05, h^2_\alpha = 0.1$

Supplementary Figure 4: Power comparison with $\rho_{\alpha\gamma} = 0.2, r = 0.8$ and $p = 1,000$. The number of replicates is 500. Note that we evaluate the type I error rate for CAUSE, GRAPPLE, cML-MA-BIC-DP, RAPS, IVW, MR-Egger, MRMix and MR-Clust using independent SNPs.

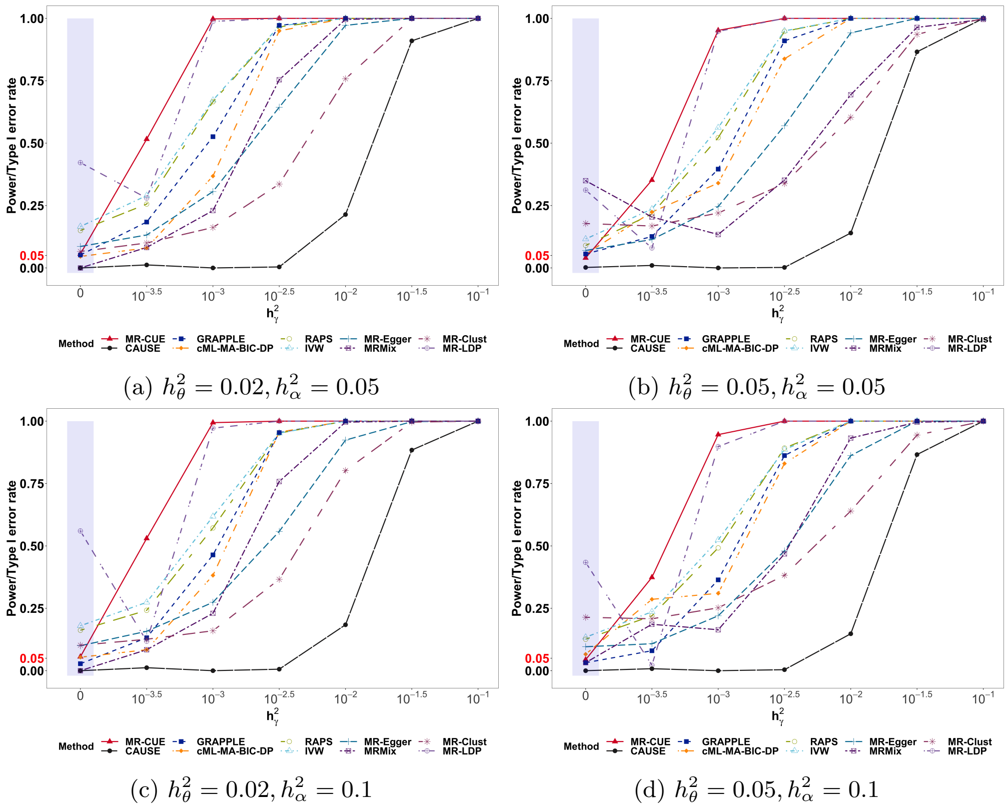

(a) $h_\theta^2 = 0.02, h_\alpha^2 = 0.05$

(b) $h_\theta^2 = 0.05, h_\alpha^2 = 0.05$

(c) $h_\theta^2 = 0.02, h_\alpha^2 = 0.1$

(d) $h_\theta^2 = 0.05, h_\alpha^2 = 0.1$

Supplementary Figure 5: Power comparison with $\rho_{\alpha\gamma} = 0.2, r = 0.4$ and $p = 2,000$. The number of replicates is 500. Note that we evaluate the type I error rate for CAUSE, GRAPPLE, cML-MA-BIC-DP, RAPS, IVW, MR-Egger, MRMix and MR-Clust using independent SNPs.

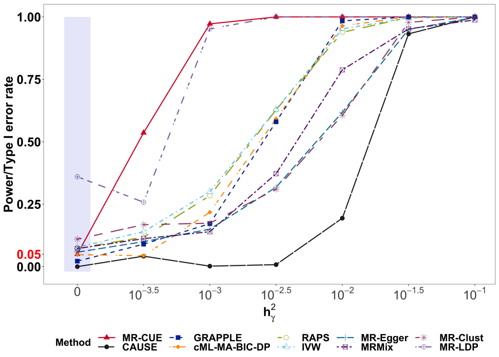

(a) $h^2_\theta = 0.02, h^2_\alpha = 0.05$

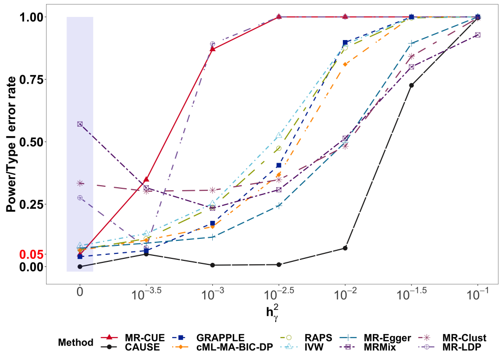

(b) $h^2_\theta = 0.05, h^2_\alpha = 0.05$

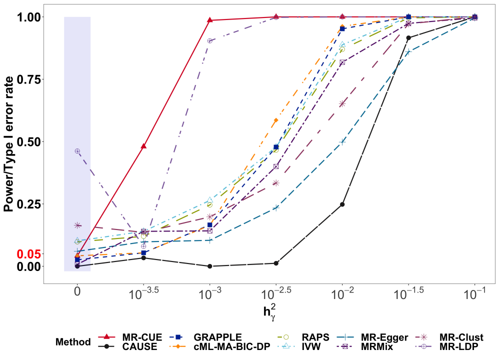

(c) $h^2_\theta = 0.02, h^2_\alpha = 0.1$

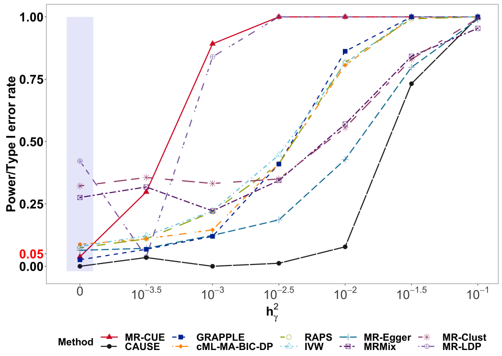

(d) $h^2_\theta = 0.05, h^2_\alpha = 0.1$

Supplementary Figure 6: Power comparison with $\rho_{\alpha\gamma} = 0.2, r = 0.8$ and $p = 2,000$. The number of replicates is 500. Note that we evaluate the type I error rate for CAUSE, GRAPPLE, cML-MA-BIC-DP, RAPS, IVW, MR-Egger, MRMix and MR-Clust using independent SNPs.

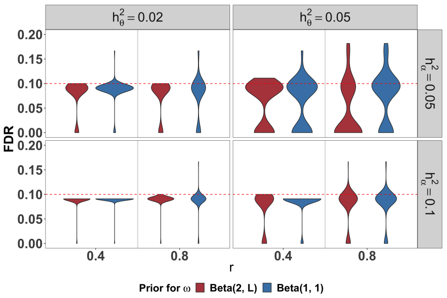

(a) $p = 1,000, \rho_{\alpha\gamma} = 0.2, \beta_1 = 0$

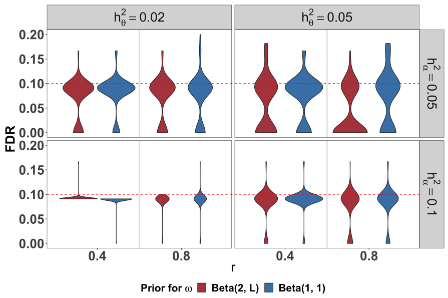

(b) $p = 1,000, \rho_{\alpha\gamma} = 0.2, \beta_1 = 0.1$

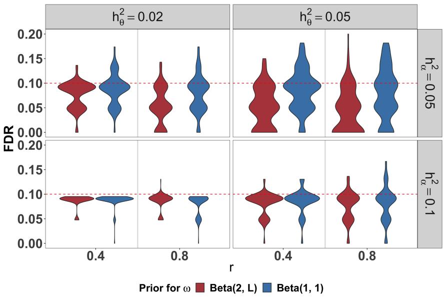

(c) $p = 2,000, \rho_{\alpha\gamma} = 0.2, \beta_1 = 0$

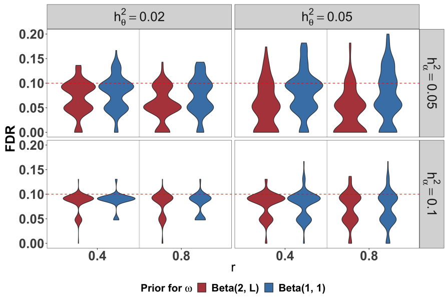

(d) $p = 2,000, \rho_{\alpha\gamma} = 0.2, \beta_1 = 0.1$

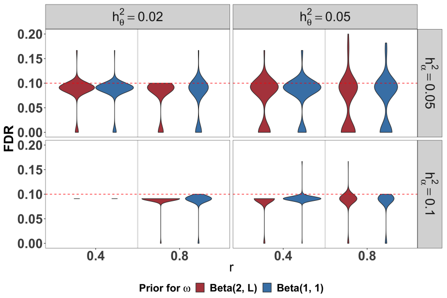

(e) $p = 1,000, \rho_{\alpha\gamma} = 0.8, \beta_1 = 0$

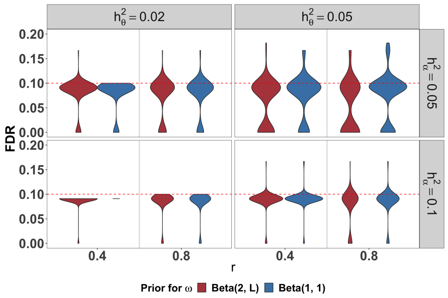

(f) $p = 1,000, \rho_{\alpha\gamma} = 0.8, \beta_1 = 0.1$

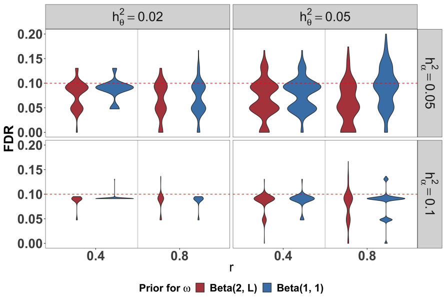

(g) $p = 2,000, \rho_{\alpha\gamma} = 0.8, \beta_1 = 0$

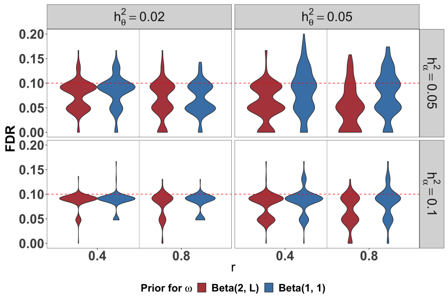

(h) $p = 2,000, \rho_{\alpha\gamma} = 0.8, \beta_1 = 0.1$

Supplementary Figure 7: Evaluation of FDR with $\beta_1 = 0$ (left panel) and $\beta_1 = 0.1$ (right panel) . The number of replicates is 100.

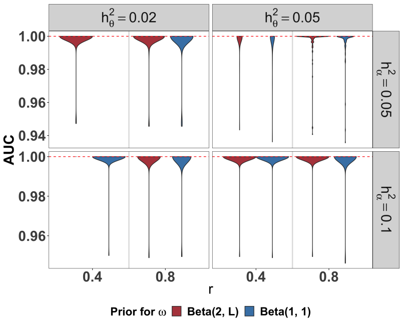

(a) $p = 1,000, \rho_{\alpha\gamma} = 0.2, \beta_1 = 0$

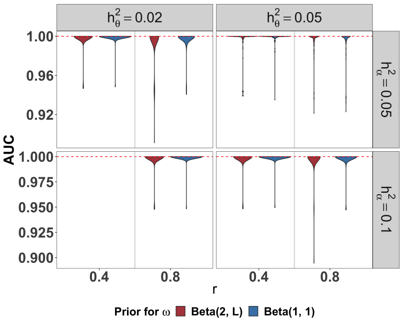

(b) $p = 1,000, \rho_{\alpha\gamma} = 0.2, \beta_1 = 0.1$

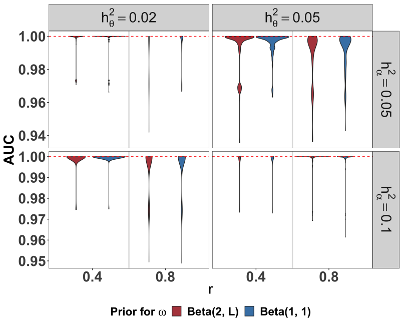

(c) $p = 2,000, \rho_{\alpha\gamma} = 0.2, \beta_1 = 0$

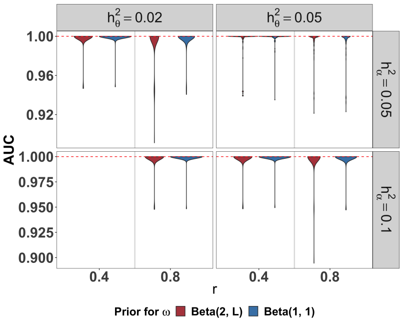

(d) $p = 1,000, \rho_{\alpha\gamma} = 0.2, \beta_1 = 0.1$

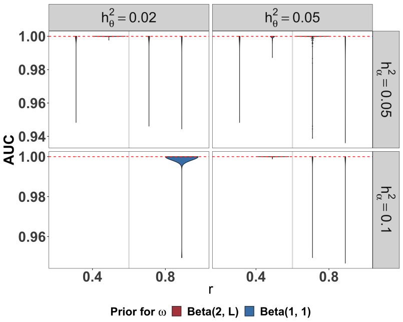

(e) $p = 1,000, \rho_{\alpha\gamma} = 0.8, \beta_1 = 0$

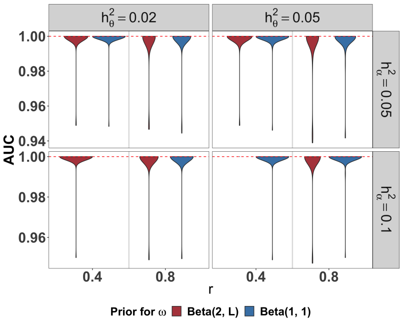

(f) $p = 1,000, \rho_{\alpha\gamma} = 0.8, \beta_1 = 0.1$

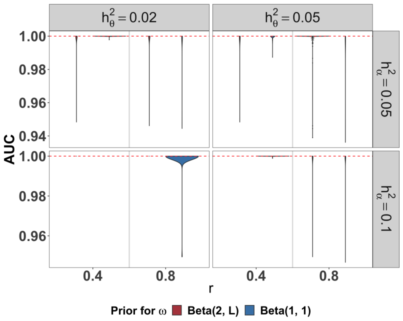

(g) $p = 2,000, \rho_{\alpha\gamma} = 0.8, \beta_1 = 0$

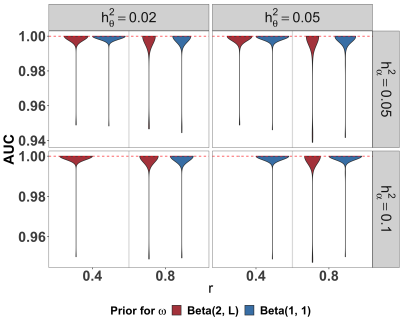

(h) $p = 2,000, \rho_{\alpha\gamma} = 0.8, \beta_1 = 0.1$

Supplementary Figure 8: Evaluation of AUC using different prior for ω with $\beta_1 = 0$ (left panel) and $\beta_1 = 0.1$ (right panel). The number of replicates is 100.

3.3 Generation of summary statistics for multiple confounding pathways

In this subsection, we introduce the detailed simulation settings related to the scenario of multiple confounding pathways. Specifically, we simulate 5 groups of IVs being associated with 5 different confounders with varying effect sizes. In details, for the l-th block, we simulate

$$
\begin{aligned}
\gamma_{lk} &\sim \mathcal{N}\left(0, \sigma_\gamma^2\right) \\
\alpha_{lk} &= \delta \cdot \gamma_{lk} + \widetilde{\alpha}_{lk}
\end{aligned}
$$

where $\widetilde{\alpha}_{lk} \sim \mathcal{N}\left(0, \sigma_\alpha^2\right)$ and $\gamma_{lk} \perp\!\!\!\perp \widetilde{\alpha}_{lk}$. The five groups of IVs are associated with those five confounders with effects, $(\sigma_{\alpha 1}^2 = 0.02, \delta_1 = 0.02)$, $(\sigma_{\alpha 2}^2 = 0.01, \delta_2 = 0.05)$, $(\sigma_{\alpha 3}^2 = 0.01, \delta_3 = 0.1)$, $(\sigma_{\alpha 4}^2 = 0.005, \delta_4 = 0.5)$, and $(\sigma_{\alpha 5}^2 = 0.005, \delta_5 = 0.8)$. We consider α_k to be sparse, i.e., only 10% of α_k's are sampled from the bivariate normal distribution and the others were zero. For UHP, we assume θ_k to be dense and follow an independent normal distribution, $\mathcal{N}(0, \sigma_\theta^2)$.

Once we have $\boldsymbol{\alpha}$, $\boldsymbol{\gamma}$ and $\boldsymbol{\theta}$, we adopte similar procedure to generate the individual-level data,

$$
\begin{aligned}
\mathbf{x}_x &= \mathbf{G}_x \boldsymbol{\gamma} + \mathbf{U}_x \boldsymbol{\psi}_x + \boldsymbol{\epsilon}_{x_x}, \\
\mathbf{x}_y &= \mathbf{G}_y \boldsymbol{\gamma} + \mathbf{U}_y \boldsymbol{\psi}_x + \boldsymbol{\epsilon}_{x_y}, \\
\mathbf{y} &= \beta_1 \mathbf{x}_y + \mathbf{G}_y \boldsymbol{\alpha} + \mathbf{G}_y \boldsymbol{\theta} + \mathbf{U}_y \boldsymbol{\psi}_y + \boldsymbol{\epsilon}_y,
\end{aligned}
$$

where $\mathbf{U}_x \in \mathbb{R}^{n_x \times q}$ and $\mathbf{U}_y \in \mathbb{R}^{n_y \times q}$ are the matrices for q confounders in the samples from IV-to-exposure and IV-to-outcome, respectively, $\boldsymbol{\psi}_x \in \mathbb{R}^{q \times 1}$ and $\boldsymbol{\psi}_y \in \mathbb{R}^{q \times 1}$ are the corresponding vector of coefficients, $\mathbf{x}_x$ and $\mathbf{x}_y$ are exposure traits in two samples,

$\boldsymbol{\epsilon}_{x_x} \in \mathbb{R}^{n_x \times 1}$, $\boldsymbol{\epsilon}_{x_y} \in \mathbb{R}^{n_y \times 1}$, and $\boldsymbol{\epsilon}_y \in \mathbb{R}^{n_y \times 1}$ are the random errors, and β_1 is the causal effect of interest. Then we generate summary statistics the same as the scenario of single confounder (see Methods).

3.4 Simulation results for non-linear confounders

For non-linear confounders, we consider the following structural model to generate individual-level data

$$\begin{aligned}
\mathbf{x}_x &= \mathbf{G}_x\boldsymbol{\gamma} + \exp(\kappa \mathbf{U}_x \boldsymbol{\psi}_x) + \boldsymbol{\epsilon}_{x_x}, \\
\mathbf{x}_y &= \mathbf{G}_y\boldsymbol{\gamma} + \exp(\kappa \mathbf{U}_y \boldsymbol{\psi}_x) + \boldsymbol{\epsilon}_{x_y}, \\
\mathbf{y} &= \beta_1 \mathbf{x}_y + \mathbf{G}_y\boldsymbol{\alpha} + \mathbf{G}_y\boldsymbol{\theta} + \exp(\kappa \mathbf{U}_y \boldsymbol{\psi}_y) + \boldsymbol{\epsilon}_y
\end{aligned}$$

where $\mathbf{U}_x \in \mathbb{R}^{n_x \times q}$ and $\mathbf{U}_y \in \mathbb{R}^{n_y \times q}$ are the matrices for q confounders for exposure and outcome, respectively. We set $\kappa = 0.1$. As shown in Fig. 9, the conclusions on the controls of type I error rates and power comparison are similar to those with linear confounders (Fig. 2 (a) and 2 (c)).

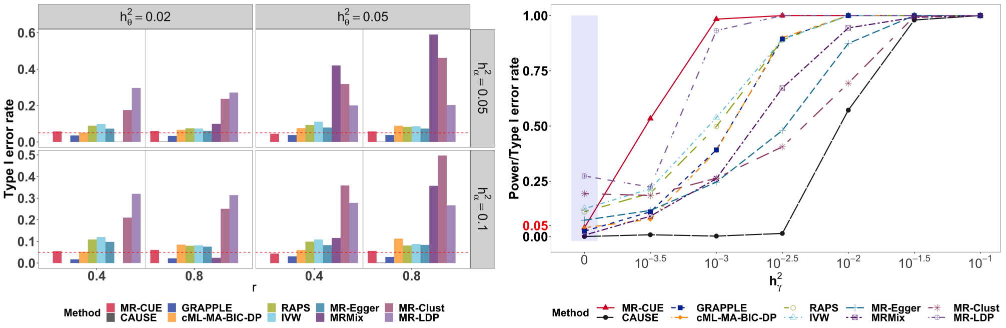

(a) Type I error rate (b) Power/Type I error rate

Supplementary Figure 9: Simulation results with non-linear confounders.(a) Type I error rates for MR-CUE and other methods under combinatorial settings for h^2_θ and h^2_α with $\rho_{\alpha\gamma} = 0.2$ and $p = 1,000$. (b) Powers for MR-CUE and other methods under the setting: $h^2_\theta = 0.02, h^2_\alpha = 0.05, p = 1,000, r = 0.4$ and $\rho_{\alpha\gamma} = 0.2$.

3.5 Simulation results for binary outcome

For binary outcome, we generate data with binary outcome using the following logistic model:

$$
\begin{aligned}
\mathbf{x}_x &= \mathbf{G}_x\boldsymbol{\gamma} + \mathbf{U}_x\boldsymbol{\psi}_x + \boldsymbol{\epsilon}_{x_x}, \\
\mathbf{x}_y &= \mathbf{G}_y\boldsymbol{\gamma} + \mathbf{U}_y\boldsymbol{\psi}_x + \boldsymbol{\epsilon}_{x_y}, \\
\mathbf{y} &= \text{Bernoulli}(H(\log(1/9) + \beta_1\mathbf{x}_y + \mathbf{G}_y\boldsymbol{\alpha} + \mathbf{G}_y\boldsymbol{\theta} + \mathbf{U}_y\boldsymbol{\psi}_y))
\end{aligned}
$$

where $H(t) = 1/(1+\exp(t))$. The population prevalence is set to be 0.1. We first generate a large population pool of outcomes and sampled 25, 000 cases and 25, 000 controls for the following analysis. As shown in Fig. 10, the conclusions on the controls of type I error rates and power comparison are similar to those with continuous outcome (Fig. 2(a) and 2(c)).

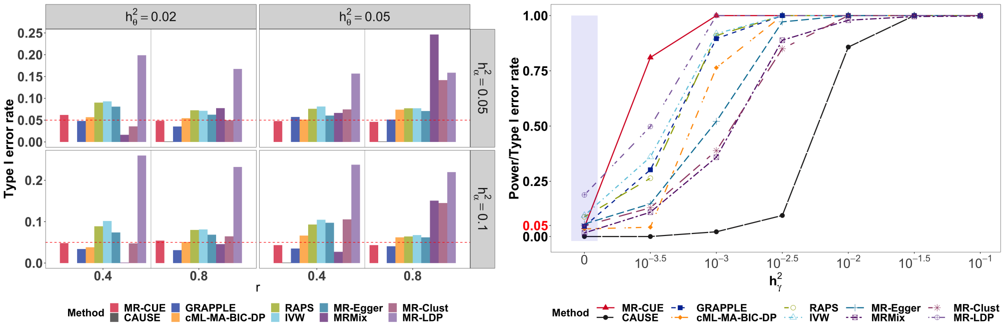

(a) Type I error rate

(b) Power/Type I error rate

Supplementary Figure 10: Simulation results with binary outcomes. (a) Type I error rates for MR-CUE and other methods under combinatorial settings for h_θ^2 and h_α^2 with $\rho_{\alpha\gamma} = 0.2$ and $p = 1,000$. (b) Powers for MR-CUE and other methods under the setting: $h_\theta^2 = 0.02, h_\alpha^2 = 0.05, p = 1,000, r = 0.4$ and $\rho_{\alpha\gamma} = 0.2$.

3.6 Simulation results for different proportions of IVs with CHP effects

We conduct additional simulations to compare with other methods when the proportions of IV with CHP effect increases. As shown in Fig. 11, when the proportion of IVs with CHP

is 0.5, the method CAUSE is conservative and very under-powered. MR-CUE, GRAPPLE and cML-MA-BIT-DP all start to show some lost in the control of type I error rates (with GRAPPLE being the worst and ours the best). MR-CUE is still the most powerful among the methods that have a reasonable control of type I error rates.

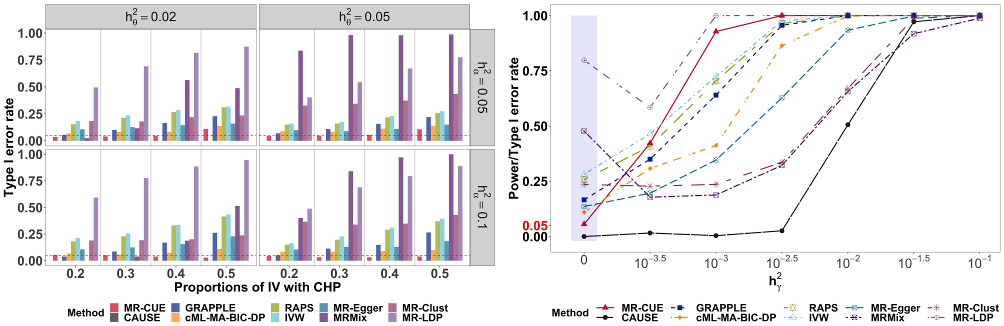

(a) Type I error rate (b) Power/Type I error rate

Supplementary Figure 11: Simulation results for different proportions of IVs with CHP effects. (a) Type I error rate comparison for MR-CUE and other methods under combinatorial settings for h_θ^2 and h_α^2 with $\rho_{\alpha\gamma} = 0.2, r = 0.4$ and $p = 1,000$. (b) Power comparison for MR-CUE and other methods under the setting: $h_\theta^2 = 0.02, h_\alpha^2 = 0.05, p = 1,000, r = 0.4, \rho_{\alpha\gamma} = 0.2$ and with the proportions of IVs with CHP effects being 0.4.

3.7 Simulation results for sparse θ in reverse causation analysis

In addition, for the reverse causation analysis, we consider a sparse vector θ, where only a small fraction of SNPs have CHP effects. In Fig. 12, we present methods comparison at different level of UHP sparsity. The conclusions are unchanged.

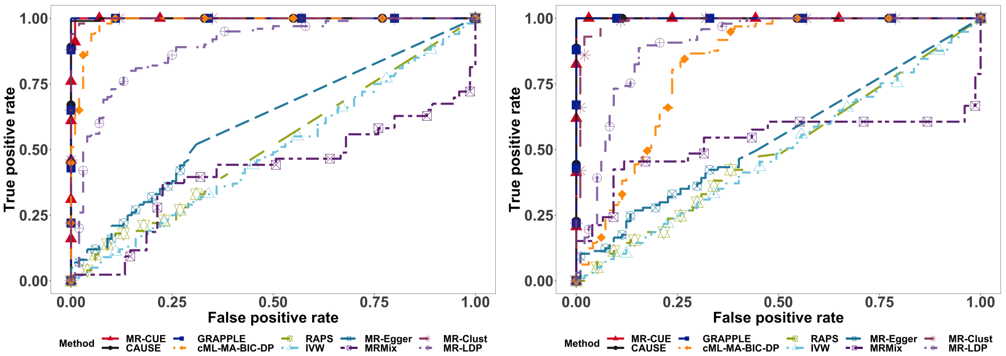

(a) Sparse rate of θ: 0.1

(b) Sparse rate of θ: 0.3

Supplementary Figure 12: The result of reverse causation with different sparse rate of θ.

Supplementary Note 4
Sensitive Analysis

4.1 Negative and Positive Controls

In this section, we adopte different p-value thresholds to select instrument variables for both negative and positive controls, the corresponding quantile-quantile plot are presented in Fig. 13-14. In addition, we display the result of negative control using correlated IVs at different IV selection thresholds, in comparison with the one using only independent IVs (labeled as MR-CUE-Indep), see Fig.15.

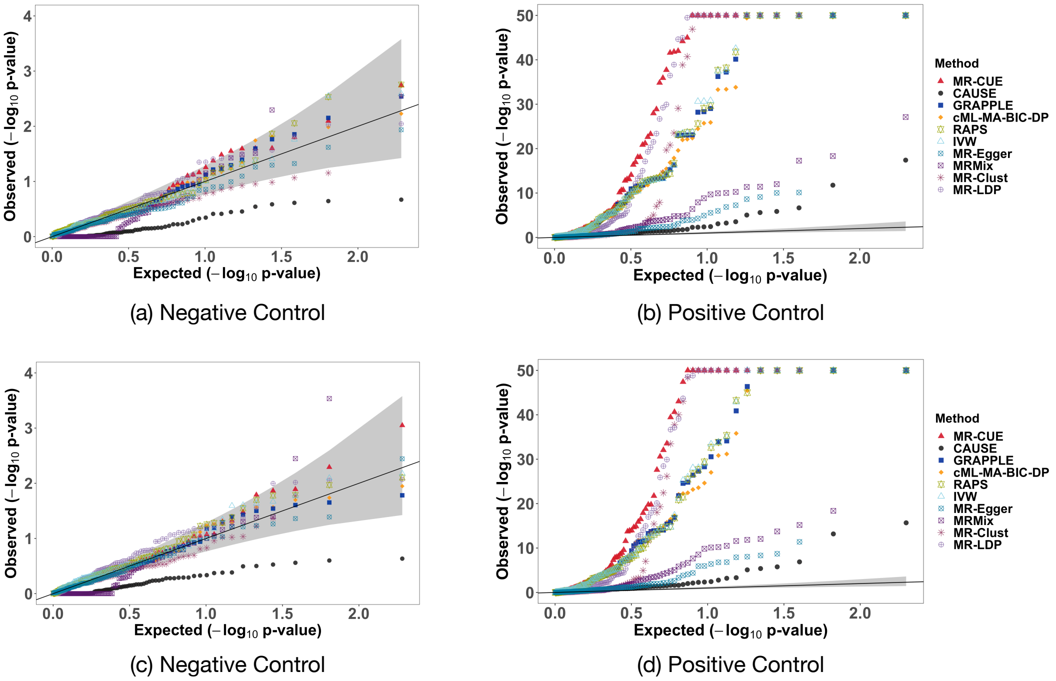

Supplementary Figure 13: The result of sensitive analysis with different IV selection thresholds, 10^{-4} (top panel) and 5×10^{-5}(bottom panel), respectively. The grey regions indicate 95% confidence intervals. p-values from all methods are two-sided without adjustment for multiple testing.

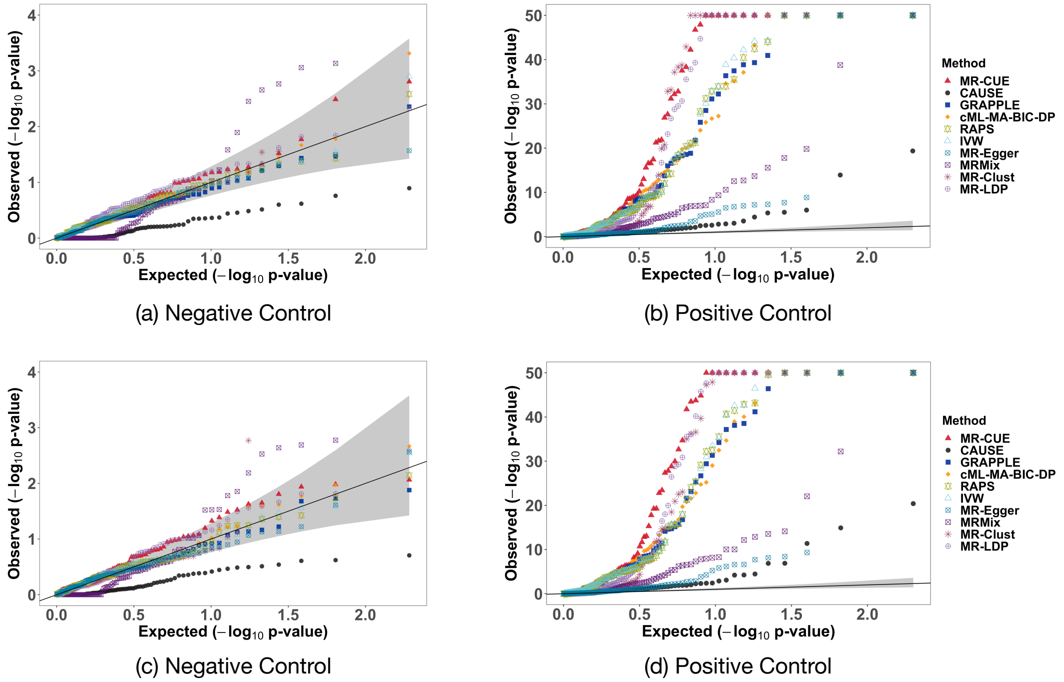

Supplementary Figure 14: The result of sensitive analysis with different IV selection thresholds, 10^{-5} (top panel) and 5×10^{-6} (bottom panel), respectively. The grey regions indicate 95% confidence intervals. p-values from all methods are two-sided without adjustment for multiple testing.

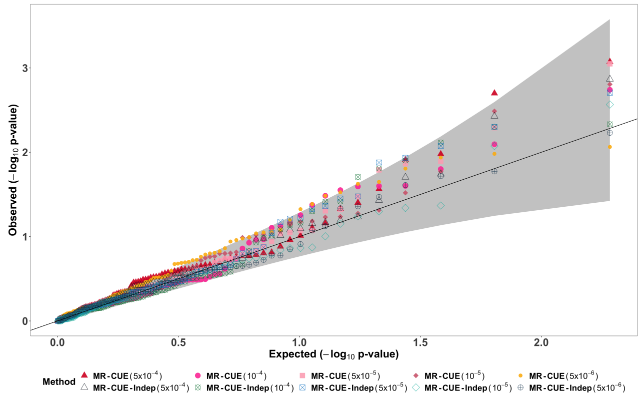

Supplementary Figure 15: The result of negative control with correlated and independent SNPs, respectively. Brackets indicate different IV selection thresholds. The grey region indicates a 95% confidence interval. p-values are two-sided without adjustment for multiple testing.

Supplementary Note 5
Real Data Analysis

The estimated causal effects of IL-6 as exposure on 27 outcome traits/disease using MR-CUE and other methods are displayed in Table 3. The estimated causal effects of T2D (European) and T2D (Asian) as outcome using MR-CUE and other methods are presented in Table 4 and Table 5, respectively.

Exposure	MR-CUE	CAUSE	GRAPPLE	cML-MA-BIC-DP	RAPS	IVW	MR-Egger	MRMix	MR-Clust	MR-LDP
SAA	**1.9×10^{-16}** ↑	0.309 ↓	0.252 ↑	0.363 ↑	0.560 ↑	0.558 ↑	0.778 ↓	0.903 ↓	0.558 ↑	**3.8×10^{-38}** ↑
HDL	**1.2×10^{-8}** ↑	0.999 ↑	0.460 ↓	0.563 ↓	0.804 ↓	0.825 ↑	0.339 ↓	0.616 ↑	0.705 ↓	**4.6×10^{-7}** ↑
LDL	**0.003** ↑	0.996 ↑	0.725 ↑	0.744 ↑	0.708 ↓	0.545 ↓	0.764 ↓	1.000 ↓	0.656 ↑	0.086 ↑
TG	**5.8×10^{-10}** ↑	0.931 ↓	0.712 ↓	0.868 ↓	0.432 ↓	0.434 ↓	0.955 ↓	0.685 ↑	0.530 ↑	**6.8×10^{-8}** ↑
TC	**1.4×10^{-4}** ↑	1.000 ↑	0.696 ↑	0.626 ↑	0.711 ↓	0.669 ↓	0.585 ↓	1.000 ↓	0.820 ↑	**0.008** ↑
COVID19Sev	**6.2×10^{-55}** ↓	0.539 ↓	0.165 ↓	0.309 ↓	0.164 ↓	0.176 ↓	**0.019** ↓	0.959 ↑	0.176 ↓	**9.3×10^{-56}** ↓
COVID19Sus	**4.8×10^{-29}** ↓	0.998 ↓	0.786 ↓	0.807 ↓	0.908 ↓	0.908 ↓	0.071 ↓	0.772 ↑	0.908 ↓	**1.2×10^{-34}** ↓
AS	**1.8×10^{-30}** ↓	0.916 ↓	0.387 ↓	0.510 ↓	0.404 ↓	0.414 ↓	0.309 ↓	0.782 ↑	0.414 ↓	**0.0×10^{0}** ↓
AIS	**1.3×10^{-38}** ↓	0.810 ↓	0.365 ↓	0.440 ↓	0.438 ↓	0.437 ↓	0.285 ↓	0.792 ↑	0.437 ↓	**0.0×10^{0}** ↓
CES	**7.0×10^{-100}** ↓	0.930 ↓	0.887 ↓	0.900 ↓	0.653 ↓	0.624 ↓	0.627 ↑	0.442 ↓	0.624 ↓	**7.0×10^{-211}** ↓
SVS	**1.0×10^{-276}** ↓	0.714 ↓	0.776 ↓	0.789 ↓	0.715 ↓	0.613 ↓	0.794 ↑	0.458 ↓	0.613 ↓	**4.9×10^{-324}** ↓
T2D	**7.1×10^{-35}** ↓	0.440 ↓	0.751 ↓	0.795 ↓	0.673 ↓	0.505 ↓	0.637 ↑	1.000 ↓	0.761 ↑	**0.035** ↑
CAD	**0.001** ↓	0.801 ↓	0.179 ↓	0.328 ↓	0.153 ↓	0.155 ↓	0.517 ↓	0.279 ↓	0.242 ↓	**1.1×10^{-9}** ↓
T1D	**1.5×10^{-53}** ↓	0.970 ↓	0.585 ↑	0.692 ↑	0.648 ↑	0.679 ↑	0.518 ↑	0.978 ↓	0.804 ↑	**3.0×10^{-69}** ↓
AD	**8.7×10^{-56}** ↑	0.845 ↓	0.274 ↓	0.469 ↓	0.270 ↓	0.303 ↓	0.212 ↓	0.939 ↓	0.303 ↓	**4.7×10^{-46}** ↑
RA	**8.7×10^{-9}** ↓	0.991 ↓	0.995 ↑	0.995 ↑	0.862 ↑	0.872 ↑	0.767 ↑	0.851 ↓	0.872 ↑	**1.2×10^{-117}** ↓
CD	**1.3×10^{-206}** ↓	0.991 ↑	0.300 ↓	0.390 ↓	0.484 ↓	0.330 ↓	**0.031** ↑	0.802 ↓	0.330 ↓	**2.6×10^{-317}** ↓
UC	**1.4×10^{-54}** ↑	0.973 ↓	0.914 ↓	0.993 ↓	0.837 ↓	0.784 ↓	0.666 ↑	0.687 ↓	0.784 ↓	**9.9×10^{-11}** ↑
Asthma	**0.049** ↑	0.984 ↑	0.419 ↑	0.461 ↑	0.257 ↑	0.212 ↑	0.922 ↓	1.000 ↓	0.378 ↑	0.469 ↑
BMD	**3.5×10^{-5}** ↓	0.427 ↓	0.625 ↓	0.742 ↓	0.270 ↓	0.586 ↓	0.182 ↓	1.000 ↓	0.299 ↑	**2.9×10^{-13}** ↓
BUN	**4.3×10^{-24}** ↑	0.639 ↑	0.078 ↑	0.141 ↑	0.130 ↑	0.142 ↑	0.218 ↑	1.000 ↓	0.188 ↑	**1.2×10^{-34}** ↑
MDD	**1.1×10^{-8}** ↓	0.130 ↓	0.079 ↓	0.245 ↓	0.075 ↓	0.185 ↓	**0.002** ↓	**0.011** ↑	0.235 ↓	**1.8×10^{-93}** ↑
BIP	**8.1×10^{-49}** ↓	1.000 ↑	0.762 ↑	0.852 ↑	0.557 ↑	0.505 ↑	0.290 ↓	0.494 ↑	0.505 ↑	**6.4×10^{-45}** ↓
SCZ	0.663 ↓	0.883 ↑	0.824 ↑	0.573 ↑	0.567 ↑	0.215 ↑	0.055 ↓	0.193 ↑	0.557 ↓	**2.8×10^{-6}** ↑
eGFR	0.413 ↑	0.996 ↓	0.133 ↑	0.249 ↑	0.589 ↑	0.732 ↑	0.155 ↑	1.000 ↓	0.203 ↑	**2.8×10^{-8}** ↓
AF	0.355 ↓	0.705 ↑	0.828 ↑	0.742 ↑	0.744 ↑	0.360 ↑	0.204 ↓	0.382 ↑	0.493 ↓	1.000 ↑
Liver	0.793 ↓	0.803 ↓	0.189 ↓	0.353 ↓	0.290 ↓	0.221 ↓	0.592 ↓	1.000 ↓	0.221 ↓	**4.7×10^{-4}** ↓

Supplementary Table 3: Summary results for evaluating the causal effects of IL-6 on 27 outcome traits/disease. Columns 2-11 listed the p-values for each MR method, p-values are marked in bold if $p < 0.05$. Arrows indicate the signs of the corresponding effect estimates. p-values from all methods are two-sided without adjustment for multiple testing.

Exposure	MR-CUE	CAUSE	GRAPPLE	cML-MA-BIC-DP	RAPS	IVW	MR-Egger	MRMix	MR-Clust	MR-LDP
HIP	**3.4×10^{-14}** ↑	0.058 ↑	**0.002** ↓	**0.001** ↓	**1.9×10^{-4}** ↑	**3.0×10^{-5}** ↑	0.321 ↑	0.271 ↓	0.809 ↓	0.204 ↓
WC	**7.6×10^{-41}** ↑	**7.5×10^{-5}** ↑	**3.7×10^{-42}** ↑	**4.5×10^{-62}** ↑	**0.000** ↑	**5.3×10^{-18}** ↑	0.709 ↑	**2.2×10^{-47}** ↑	**0.000** ↑	**1.1×10^{-46}** ↑
WHR	**6.1×10^{-14}** ↑	**2.1×10^{-4}** ↑	**2.6×10^{-24}** ↑	**1.8×10^{-29}** ↑	**3.7×10^{-14}** ↑	**1.7×10^{-27}** ↑	0.286 ↓	**6.6×10^{-7}** ↑	0.067 ↓	**4.9×10^{-35}** ↑
BMI	**8.8×10^{-99}** ↑	**8.6×10^{-4}** ↑	**1.2×10^{-51}** ↑	**6.0×10^{-115}** ↑	**0.000** ↑	**1.8×10^{-25}** ↑	**0.023** ↑	**1.0×10^{-65}** ↑	**0.000** ↑	**2.4×10^{-112}** ↑
BFP	**2.1×10^{-10}** ↑	0.291 ↑	0.179 ↑	0.530 ↑	0.117 ↑	0.336 ↑	0.727 ↓	**2.9×10^{-50}** ↑	0.977 ↑	**1.4×10^{-30}** ↑
BL	**1.1×10^{-11}** ↓	0.936 ↓	0.104 ↓	0.135 ↓	0.102 ↓	0.112 ↓	0.686 ↓	0.320 ↓	0.172 ↑	**2.3×10^{-23}** ↓
BW	**2.9×10^{-4}** ↓	0.200 ↓	**2.0×10^{-4}** ↓	**7.4×10^{-4}** ↓	**5.0×10^{-4}** ↓	**2.0×10^{-6}** ↓	0.449 ↓	**5.7×10^{-6}** ↓	**0.015** ↑	**1.6×10^{-56}** ↓
HR	**1.2×10^{-14}** ↑	0.702 ↓	0.392 ↓	0.417 ↓	0.508 ↓	0.516 ↓	0.865 ↓	1.000 ↓	0.564 ↓	**0.009** ↓
HF	**2.3×10^{-202}** ↑	0.212 ↑	**1.7×10^{-22}** ↑	**1.5×10^{-22}** ↑	**0.000** ↑	**7.8×10^{-29}** ↑	0.512 ↑	**1.2×10^{-4}** ↑	0.298 ↓	**0.000** ↑
HDL-C	**1.0×10^{-13}** ↓	**0.027** ↓	**8.6×10^{-15}** ↓	**6.9×10^{-13}** ↓	**2.2×10^{-16}** ↓	**1.8×10^{-18}** ↓	0.497 ↓	0.114 ↓	**0.034** ↑	**5.6×10^{-73}** ↓
TG	0.950 ↑	**0.022** ↑	**1.0×10^{-13}** ↑	**5.8×10^{-17}** ↑	**3.8×10^{-14}** ↑	**1.3×10^{-18}** ↑	0.320 ↓	**0.005** ↑	**0.047** ↓	**1.9×10^{-24}** ↑
SBP	**0.000** ↑	**0.011** ↑	**2.1×10^{-21}** ↑	**3.0×10^{-21}** ↑	**0.000** ↑	**3.4×10^{-23}** ↑	**0.015** ↑	**7.2×10^{-4}** ↑	**0.002** ↓	**0.000** ↑
DBP	**0.000** ↑	**0.038** ↑	**4.1×10^{-9}** ↑	**6.0×10^{-8}** ↑	**8.9×10^{-10}** ↑	**6.2×10^{-10}** ↑	**0.041** ↑	0.166 ↑	0.552 ↓	**0.000** ↑
PP	**0.000** ↑	**0.007** ↑	**8.3×10^{-23}** ↑	**5.2×10^{-24}** ↑	**0.000** ↑	**4.7×10^{-29}** ↑	**0.003** ↑	**1.3×10^{-9}** ↑	0.186 ↓	**0.000** ↑
AIS	**4.4×10^{-8}** ↑	0.616 ↑	**4.9×10^{-11}** ↑	**3.1×10^{-10}** ↑	**7.1×10^{-13}** ↑	**2.0×10^{-14}** ↑	0.102 ↑	**0.008** ↑	0.072 ↓	**8.1×10^{-299}** ↑
MDD	**0.000** ↑	0.146 ↑	**2.2×10^{-13}** ↑	**1.1×10^{-14}** ↑	**1.5×10^{-11}** ↑	**5.6×10^{-14}** ↑	0.119 ↑	**4.9×10^{-4}** ↑	0.861 ↑	**0.000** ↑
UA	**1.0×10^{-13}** ↑	0.956 ↑	**0.004** ↑	**0.002** ↑	**0.001** ↑	**4.3×10^{-5}** ↑	0.475 ↓	0.111 ↑	0.489 ↓	**6.0×10^{-18}** ↑
RBC	**3.6×10^{-46}** ↑	0.913 ↑	**0.045** ↑	**0.004** ↑	0.151 ↑	**0.001** ↑	0.103 ↓	**0.017** ↑	**0.007** ↓	**2.5×10^{-134}** ↑
WBC	**1.2×10^{-93}** ↑	0.931 ↑	**1.1×10^{-5}** ↑	**6.7×10^{-6}** ↑	**3.5×10^{-5}** ↑	**2.6×10^{-8}** ↑	0.845 ↓	**0.016** ↑	**0.034** ↓	**9.7×10^{-172}** ↑
PLT	**6.8×10^{-34}** ↑	0.995 ↓	0.828 ↓	0.892 ↑	0.392 ↓	0.510 ↓	0.488 ↓	0.360 ↑	0.862 ↓	**3.4×10^{-28}** ↓
Glu	0.196 ↓	0.851 ↑	**0.029** ↑	0.103 ↑	**0.021** ↑	**0.009** ↑	0.805 ↑	0.680 ↓	0.629 ↑	**5.6×10^{-17}** ↑
Gly	**2.3×10^{-8}** ↑	0.699 ↑	0.884 ↑	0.788 ↑	0.979 ↓	0.928 ↑	0.986 ↓	0.412 ↑	0.748 ↓	**8.8×10^{-9}** ↓
DHA	**6.3×10^{-59}** ↓	1.000 ↑	0.110 ↑	0.119 ↑	0.114 ↑	0.178 ↑	0.057 ↑	0.561 ↑	0.092 ↑	**3.6×10^{-5}** ↑
OA	**1.2×10^{-18}** ↑	1.000 ↑	0.117 ↑	0.239 ↑	0.188 ↑	0.335 ↑	0.242 ↑	**3.5×10^{-4}** ↓	0.302 ↑	1.000 ↓
ISI	**3.5×10^{-4}** ↓	0.423 ↓	0.575 ↓	0.972 ↓	0.189 ↓	0.165 ↓	0.850 ↓	0.236 ↑	0.697 ↑	**1.7×10^{-18}** ↓
FG	**7.0×10^{-5}** ↑	**0.007** ↑	0.269 ↑	0.071 ↑	**8.5×10^{-5}** ↑	**2.8×10^{-4}** ↑	0.099 ↓	**1.4×10^{-5}** ↑	0.471 ↓	**1.6×10^{-141}** ↑
FI	**1.5×10^{-69}** ↑	0.056 ↑	**2.6×10^{-4}** ↑	**0.005** ↑	0.199 ↑	**3.6×10^{-4}** ↑	0.686 ↓	0.084 ↑	0.201 ↓	**3.1×10^{-12}** ↑
IR	**5.0×10^{-211}** ↑	0.146 ↑	**1.1×10^{-7}** ↑	**3.5×10^{-7}** ↑	**2.8×10^{-8}** ↑	**1.7×10^{-10}** ↑	0.904 ↓	**8.1×10^{-4}** ↑	0.169 ↑	**3.3×10^{-96}** ↑
HbA1c	**2.7×10^{-186}** ↑	0.394 ↑	**2.1×10^{-10}** ↑	**2.1×10^{-9}** ↑	**4.0×10^{-15}** ↑	**3.1×10^{-18}** ↑	0.653 ↓	**4.0×10^{-4}** ↑	0.062 ↓	**4.3×10^{-117}** ↑

Supplementary Table 4: Summary results for evaluating the causal effects of 29 exposures on T2D risk in the European population. Columns 2-11 listed the p-values for each MR method, p-values are marked in bold if $p < 0.05$. Arrows indicate the signs of the corresponding effect estimates. p-values from all methods are two-sided without adjustment for multiple testing.

Exposure	MR-CUE	CAUSE	GRAPPLE	cML-MA-BIC-DP	RAPS	IVW	MR-Egger	MRMix	MR-Clust	MR-LDP
BMI	**2.1×10^{-27}** ↑	0.076 ↑	**3.7×10^{-8}** ↑	**2.3×10^{-16}** ↑	0.066 ↑	0.089 ↑	0.050 ↑	**2.9×10^{-11}** ↑	**3.3×10^{-6}** ↓	**8.2×10^{-51}** ↑
HF	**0.004** ↑	0.992 ↑	**0.001** ↑	**0.005** ↑	**8.6×10^{-4}** ↑	**0.001** ↑	0.437 ↑	0.103 ↑	0.165 ↑	0.213 ↑
HDL-C	**3.8×10^{-11}** ↓	1.000 ↓	**0.038** ↓	**0.009** ↓	**0.037** ↓	**0.007** ↓	**0.042** ↑	0.055 ↓	0.567 ↑	**4.3×10^{-4}** ↓
TG	**1.8×10^{-36}** ↑	0.586 ↑	**1.0×10^{-4}** ↑	**5.9×10^{-5}** ↑	**0.006** ↑	**0.012** ↑	**0.006** ↓	**0.002** ↑	0.142 ↓	**0.001** ↑
SBP	0.209 ↑	0.145 ↑	**2.1×10^{-6}** ↑	**3.4×10^{-6}** ↑	**1.3×10^{-5}** ↑	**2.1×10^{-5}** ↑	0.653 ↑	**0.003** ↑	0.066 ↑	**6.6×10^{-9}** ↑
DBP	**0.003** ↑	1.000 ↓	0.249 ↑	0.270 ↑	0.814 ↓	0.591 ↓	0.807 ↓	0.274 ↑	0.708 ↓	0.188 ↑
PP	**7.6×10^{-23}** ↑	0.837 ↑	**0.018** ↑	**0.025** ↑	**0.009** ↑	**0.010** ↑	0.435 ↑	0.211 ↑	0.529 ↑	**1.6×10^{-27}** ↑
AIS	**1.2×10^{-5}** ↑	0.331 ↑	**3.1×10^{-4}** ↑	**5.6×10^{-4}** ↑	**4.8×10^{-4}** ↑	**1.7×10^{-4}** ↑	0.899 ↓	**0.026** ↑	0.629 ↑	**8.7×10^{-11}** ↑
RBC	**4.0×10^{-23}** ↑	0.184 ↑	**1.3×10^{-7}** ↑	**5.5×10^{-6}** ↑	**4.2×10^{-6}** ↑	**9.6×10^{-7}** ↑	0.740 ↑	**2.6×10^{-5}** ↑	0.462 ↑	**1.4×10^{-45}** ↓
WBC	**2.5×10^{-36}** ↑	0.510 ↑	**2.0×10^{-4}** ↑	**2.5×10^{-4}** ↑	**2.5×10^{-5}** ↑	**6.0×10^{-5}** ↑	0.676 ↑	**0.010** ↑	0.424 ↑	**4.3×10^{-6}** ↑
PLT	0.603 ↑	0.993 ↓	0.071 ↓	**0.040** ↓	**0.022** ↓	**0.038** ↓	0.421 ↓	0.142 ↓	0.145 ↓	**1.1×10^{-13}** ↑
Glu	**4.3×10^{-5}** ↓	0.930 ↓	0.417 ↓	0.489 ↓	0.307 ↓	0.337 ↓	0.462 ↓	1.000 ↓	0.529 ↓	**8.2×10^{-32}** ↑
Gly	**3.3×10^{-13}** ↑	1.000 ↑	0.462 ↓	0.508 ↓	0.809 ↓	0.906 ↓	0.258 ↓	0.705 ↑	0.679 ↓	**6.2×10^{-68}** ↓
HbA1c	**4.8×10^{-4}** ↑	0.990 ↑	0.403 ↑	0.648 ↑	0.145 ↑	0.132 ↑	0.215 ↑	0.479 ↓	0.408 ↓	1.000 ↑

Supplementary Table 5: Summary results for evaluating the causal effects of 14 exposures on T2D risk in the East Asian population. Columns 2-11 listed the p-values for each MR method, p-values are marked in bold if $p < 0.05$. Arrows indicate the signs of the corresponding effect estimates. p-values from all methods are two-sided without adjustment for multiple testing.

Supplementary References

[1] Morrison, J., Knoblauch, N., Marcus, J. H., Stephens, M. & He, X. Mendelian randomization accounting for correlated and uncorrelated pleiotropic effects using genome-wide summary statistics. *Nature genetics* **52**, 740–747 (2020).

[2] Xue, H., Shen, X. & Pan, W. Constrained maximum likelihood-based mendelian randomization robust to both correlated and uncorrelated pleiotropic effects. *The American Journal of Human Genetics* **108**, 1251–1269 (2021).

[3] Bowden, J., Davey Smith, G., Haycock, P. C. & Burgess, S. Consistent estimation in mendelian randomization with some invalid instruments using a weighted median estimator. *Genetic epidemiology* **40**, 304–314 (2016).

[4] Verbanck, M., Chen, C.-Y., Neale, B. & Do, R. Detection of widespread horizontal pleiotropy in causal relationships inferred from mendelian randomization between complex traits and diseases. *Nature genetics* **50**, 693–698 (2018).
