# Supplementary material for: Mendelian randomization accounting for complex correlated horizontal pleiotropy while elucidating shared genetic etiology
Source: Nat Commun. 2022 Oct 30;13:6490. doi: 10.1038/s41467-022-34164-1 (PMC9618026; doi:10.1038/s41467-022-34164-1)
Supplement: Supplementary file 3 — Description of Additional Supplementary Files [file 41467_2022_34164_MOESM3_ESM.pdf]

Description of Additional Supplementary Files

File Name: Supplementary Data 1
Description: Study information and download links for data used.

File Name: Supplementary Data 2
Description: Related to Supplementary Tables 3-5. The estimated causal effects and corresponding standard errors using MR-CUE and other MR methods.
