# Supplementary material for: Mendelian randomization accounting for complex correlated horizontal pleiotropy while elucidating shared genetic etiology
Source: Nat Commun. 2022 Oct 30;13:6490. doi: 10.1038/s41467-022-34164-1 (PMC9618026; doi:10.1038/s41467-022-34164-1)
Supplement: Supplementary file 6 — Reporting Summary [file 41467_2022_34164_MOESM6_ESM.pdf]

Corresponding author(s): Lin S. Chen, Jin Liu

Last updated by author(s): Oct 6, 2022

Reporting Summary

Nature Portfolio wishes to improve the reproducibility of the work that we publish. This form provides structure for consistency and transparency in reporting. For further information on Nature Portfolio policies, see our Editorial Policies and the Editorial Policy Checklist.

Statistics

For all statistical analyses, confirm that the following items are present in the figure legend, table legend, main text, or Methods section.

n/a	Confirmed	
☐	☒	The exact sample size (*n*) for each experimental group/condition, given as a discrete number and unit of measurement
☒	☐	A statement on whether measurements were taken from distinct samples or whether the same sample was measured repeatedly
☐	☒	The statistical test(s) used AND whether they are one- or two-sided<br>*Only common tests should be described solely by name; describe more complex techniques in the Methods section.*
☒	☐	A description of all covariates tested
☐	☒	A description of any assumptions or corrections, such as tests of normality and adjustment for multiple comparisons
☐	☒	A full description of the statistical parameters including central tendency (e.g. means) or other basic estimates (e.g. regression coefficient) AND variation (e.g. standard deviation) or associated estimates of uncertainty (e.g. confidence intervals)
☐	☒	For null hypothesis testing, the test statistic (e.g. *F*, *t*, *r*) with confidence intervals, effect sizes, degrees of freedom and *P* value noted<br>*Give P values as exact values whenever suitable.*
☐	☒	For Bayesian analysis, information on the choice of priors and Markov chain Monte Carlo settings
☐	☒	For hierarchical and complex designs, identification of the appropriate level for tests and full reporting of outcomes
☐	☒	Estimates of effect sizes (e.g. Cohen's *d*, Pearson's *r*), indicating how they were calculated

Our web collection on statistics for biologists contains articles on many of the points above.

Software and code

Policy information about availability of computer code

Data collection	For reference panel data, we use PLINK (Version 1.90 b6.1 64-bit) and GCTA (Version 1.93.0beta) to complete the quality control process.
Data analysis	All data was analyzed using R >=3.5.0.<br>The MR-CUE method is implemented in an open-source, publicly available R package that is available at https://github.com/QingCheng0218/MR.CUE. The code to reproduce the analysis can be found at https://github.com/QingCheng0218/MR.CUE/tree/main/simulation.<br><br>Additional R packages were used for running numerical studies. These are:<br>CAUSE: Version 1.0.0 https://github.com/jean997/cause<br>GRAPPLE: https://github.com/jingshuw/GRAPPLE<br>cML-MA: https://github.com/xue-hr/MRcML<br>RAPS: https://github.com/qingyuanzhao/mr.raps<br>MendelianRandomization for IVW and MR-Egger: Version 0.4.3 https://cran.r-project.org/src/contrib/Archive/MendelianRandomization/<br>MRMix: https://github.com/gqi/MRMix<br>MR-Clust: https://github.com/cnfoley/mrclust<br>MR-LDP: https://github.com/QingCheng0218/MR.LDP

For manuscripts utilizing custom algorithms or software that are central to the research but not yet described in published literature, software must be made available to editors and reviewers. We strongly encourage code deposition in a community repository (e.g. GitHub). See the Nature Portfolio guidelines for submitting code & software for further information.

Data

Policy information about availability of data

All manuscripts must include a data availability statement. This statement should provide the following information, where applicable:

- Accession codes, unique identifiers, or web links for publicly available datasets
- A description of any restrictions on data availability
- For clinical datasets or third party data, please ensure that the statement adheres to our policy

The reference panel is the merged genotype data from UK10K and 1000 Genome Project Phase 3, available for download from the European Genome-Phenome Archive (https://www.ebi.ac.uk/ega/) with ID EGAD00001000776. The LD estimates using UK10K genotype data for the list of SNPs from HapMap Project Phase 3 (HapMap3) can be download at https://zenodo.org/record/7152063.
All GWAS summary statistics used in this study are publicly available. GWAS summary statistics for IL-6 are available at http://www.phpc.cam.ac.uk/ceu/proteins/. GWAS summary statistics for T2D in the European population can be obtained at http://diagram-consortium.org/downloads.html. GWAS summary statistics for T2D in the East Asian population can be accessed here https://blog.nus.edu.sg/agen/summary-statistics/t2d-2020/. Other summary statistics are publicly available from the studies as referenced in Supplementary Data 1.

Human research participants

Policy information about studies involving human research participants and Sex and Gender in Research.

Reporting on sex and gender	NA
Population characteristics	NA
Recruitment	NA
Ethics oversight	NA

Note that full information on the approval of the study protocol must also be provided in the manuscript.

Field-specific reporting

Please select the one below that is the best fit for your research. If you are not sure, read the appropriate sections before making your selection.

☒ Life sciences ☐ Behavioural & social sciences ☐ Ecological, evolutionary & environmental sciences

For a reference copy of the document with all sections, see nature.com/documents/nr-reporting-summary-flat.pdf

Life sciences study design

All studies must disclose on these points even when the disclosure is negative.

Sample size	The work described in this manuscript uses public summary statistics, therefore, we do not have control over the sample size. Instead, sample sizes were reported in the GWAS publications or their summary statistics, e.g., sample size of IL-6: http://www.phpc.cam.ac.uk/ceu/proteins/ , sample size of T2D in European population: sample size for http://diagram-consortium.org/downloads.html, sample size of T2D in East Asian population: https://blog.nus.edu.sg/agen/summary-statistics/t2d-2020/. We list the sample sizes for each summary statistics related in this study in Supplementary Data 1.
Data exclusions	We conducted strict quality control for the reference data using PLINK and GCTA. We removed the individuals with genotype missing rates greater than 5%, and further removed one pair of individuals that have genetic relatedness larger than 0.05. Since both ALSPAC and TwinsUK cohorts contain non-European samples, we further performed the principal components analysis followed to extract and restrict the analysis to samples from European ancestries. After data pre-processing, roughly 3,700 samples were retained as the reference panel data.
Replication	We applied MR-CUE to each exposure-T2D trait pair and separately estimated the causal effect from each exposure on T2D risk in the European and East Asian populations. The cross-populations analyses gave us additional evidence of examining multiple potential risk exposures for T2D in independent samples. All results presented in the paper can be replicated with code available on our website https://github.com/QingCheng0218/MR.CUE.
Randomization	We did not perform any randomized experiments that involves assigning individuals to groups.
Blinding	We did not perform any experiments that involves assigning individuals to groups.

Reporting for specific materials, systems and methods

We require information from authors about some types of materials, experimental systems and methods used in many studies. Here, indicate whether each material, system or method listed is relevant to your study. If you are not sure if a list item applies to your research, read the appropriate section before selecting a response.

Materials & experimental systems

n/a	Involved in the study
☒	☐ Antibodies
☒	☐ Eukaryotic cell lines
☒	☐ Palaeontology and archaeology
☒	☐ Animals and other organisms
☒	☐ Clinical data
☒	☐ Dual use research of concern

Methods

n/a	Involved in the study
☒	☐ ChIP-seq
☒	☐ Flow cytometry
☒	☐ MRI-based neuroimaging
